# *GFFx*: A Rust-based suite of utilities for ultra-fast genomic feature extraction

**DOI:** 10.1093/gigascience/giaf124

**Published:** 2025-10-23

**Authors:** Baohua Chen, Dongya Wu, Guojie Zhang

**Affiliations:** School of Basic Medical Sciences, Zhejiang University School of Medicine, Hangzhou 310058, China; Center for Evolutionary & Organismal Biology, Liangzhu Laboratory, Zhejiang University Medical Center, Hangzhou 311121, China; School of Basic Medical Sciences, Zhejiang University School of Medicine, Hangzhou 310058, China; Center for Evolutionary & Organismal Biology, Liangzhu Laboratory, Zhejiang University Medical Center, Hangzhou 311121, China; School of Basic Medical Sciences, Zhejiang University School of Medicine, Hangzhou 310058, China; Center for Evolutionary & Organismal Biology, Liangzhu Laboratory, Zhejiang University Medical Center, Hangzhou 311121, China

**Keywords:** GFF file, genome annotation, Rust programming, feature extraction

## Abstract

**Background:**

Genome annotations have become increasingly complex with the discovery of diverse regulatory elements and transcript variants, posing growing challenges for efficient data querying and storage. Existing tools often show performance bottlenecks when processing large-scale annotation files, especially for region-based searches and hierarchical feature extraction. Leveraging Rust’s advantages in execution speed, memory safety, and multithreading offers a promising path toward scalable solutions for genome annotation access.

**Findings:**

We present *GFFx*, a Rust-based toolkit for high-performance access to GFF annotation files. It employs a compact, model-aware indexing system and memory-mapped I/O to enable fast random access with minimal overhead. Benchmarks across multiple genomes show 10–80 times faster ID-based extraction, 20–60 times faster region retrieval, and 7–14 times faster coverage profiling than existing tools, while maintaining low memory use and small index size.

**Conclusions:**

*GFFx* offers a lightweight and scalable infrastructure for efficient genome annotation access and quantitative analysis. By combining Rust’s performance and safety with an extensible design, it provides a robust foundation for large-scale and multi-omics workflows.

## Introduction

With the growing understanding of functional genome regions beyond conventional protein-coding genes, genome annotations are rapidly increasing in both complexity and volume. Large-scale efforts such as ENCODE [1], FANTOM [2], and the Roadmap Epigenomics Program [3] have cataloged diverse noncoding elements—including enhancers, promoters, long noncoding RNAs (lncRNAs), and epigenetic marks—highlighting their roles in gene regulation, chromatin dynamics, and cellular identity. As novel regulatory elements, alternative isoforms, and lineage- or tissue-specific transcripts continue to emerge, annotation datasets are expected to expand further [4]. The accumulation of such multilayered annotations, particularly across large genomes or pangenomes, poses growing challenges for storage, indexing, and efficient querying.

However, existing tools often struggle to process ultra-large annotation files efficiently, particularly for region-based queries, hierarchical model extraction, or parallel execution. A scalable, high-performance solution optimized for such tasks is urgently needed. Rust, a modern systems programming language, offers high execution speed, memory safety, efficient multithreading, and cross-platform portability. These features have led to its increasing adoption in bioinformatics [5], as exemplified by Rust-Bio [6], Bigtools [7], Phylo-rs [8], and fibertools [9].

To address these challenges, we developed *GFFx*, a Rust-based toolkit for fast and scalable access to genome annotation files. *GFFx* supports region-, identity-, and attribute-based queries over ultra-large General Feature Format (GFF) datasets. Designed as both a command-line tool and a reusable library, it can be integrated into larger pipelines and software systems. It also demonstrates Rust’s potential in computational biology by providing a robust, extensible foundation for high-performance annotation processing.

## Findings

### Performance benchmark in annotation indexing

**Figure 1:**
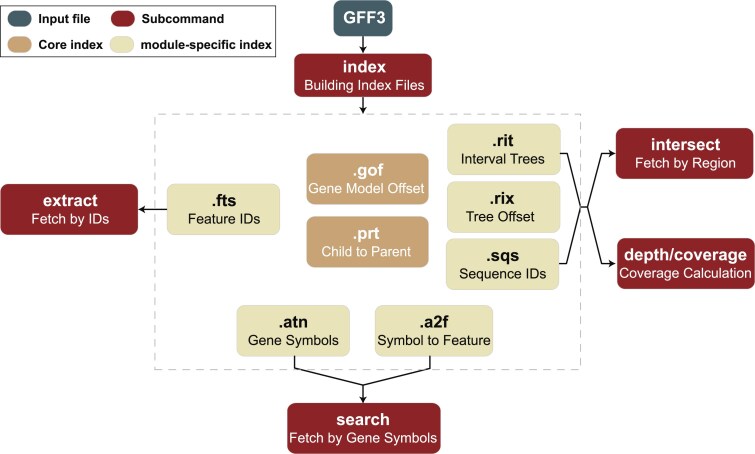
Architecture of the indexing system and subcommand interactions in *GFFx*. All index files are generated in advance from a GFF3 file (cream box) via the index module. While all subcommands (green boxes) have access to the complete set of indices, each subcommand loads only the subset relevant to its specific function. Core indices *.gof* and *.prt* (dark brown boxes) are universally required, whereas module-specific indices, including *.fts, .a2f, .atn, .rit, .rix*, and *.sqs* (light brown boxes), are utilized only by specific subcommands as illustrated.

*GFFx* achieves high-performance efficiency through a modular indexing system anchored by 2 core indices, .prt and .gof, which capture feature hierarchical relationship and map annotation blocks to their byte-offsets for direct memory access, respectively. Complementary lightweight indices, including *.fts, .a2f, .atn, .sqs, rit*, and *.rix*, support subcommand-specific operations like feature extraction, attribute-based searches, and region queries with minimal input/output (I/O) overhead (Fig. 1).

Among commonly used GFF processing tools, only *gffutils* [10] performs preprocessing by converting GFF files into an SQLite database. In contrast, *GFFx* adopts a lightweight index strategy optimized for direct file-based access. To assess the relative efficiency of these 2 approaches, we compared the runtime required for index construction in *GFFx* versus database creation in *gffutils* (v0.13).

For this evaluation, we selected 8 representative GFF3 annotation datasets spanning a broad taxonomic range and varying annotation complexities, with file sizes ranging from 156.86 to 1,511.79 MB (Supplementary Table S1). The datasets included the vertebrate genomes of *Pungitius sinensis* (ceob_ps_1.0), *Gallus gallus* (GRCg7b), *Mus musculus* (GRCm39), *Sus scrofa* (Sscrofa11.1), and *Homo sapiens* genome (hg38), as well as the invertebrate genome of *Drosophila melanogaster* (dm6) and 2 plant genomes, *Triticum aestivum* (IWGSC CS refseq v2.1) and *Arabidopsis thaliana* (Tair10.1). These datasets collectively capture the diversity of genome sizes and annotation scales observed in contemporary genomics. All benchmarks were performed on a dedicated compute node equipped with 2× Intel Xeon Gold 6448H CPUs (32 cores/64 threads each), 1 TB DDR4 RAM, and dual Micron 7450 MTFDKCB960TFR NVMe SSDs (total capacity 1.92 TB). Despite its relatively complex indexing architecture, *GFFx* consistently outperformed *gffutils*, achieving speedups of 5.81- to 8.45-fold (Supplementary Fig. S1a). This improvement was accompanied by higher memory usage. For the largest dataset hg38, *GFFx* required 2.77 GB of memory, which remains manageable on most modern computing platforms, including personal computers (Supplementary Fig. S1b). In addition, the sizes of the index files produced by both tools scaled linearly with dataset size, and the indexes generated by *GFFx* were about 2.5% to 4.1% of the size of those produced by *gffutils* (Supplementary Tables S2, S3), underscoring another key advantage of *GFFx*.

To assess the effect of dataset size within a single organism, we down-sampled hg38 and repeated the benchmarks. Runtime increased with dataset size for both tools, and *GFFx* consistently finished in about one-sixth to one-seventh of the time required by *gffutils* (Supplementary Fig. S1c). Memory usage for *GFFx* increased nearly linearly with dataset size, whereas *gffutils* remained almost constant (Supplementary Fig. S1d). Within hg38, these results indicate size-driven scaling with a stable relative advantage of *GFFx*. In contrast, cross-organism comparisons show more variability in the relative speedup, which is more plausibly explained by differences in annotation complexity, such as the density of noncoding RNAs, the prevalence of alternative splicing, and the abundance of repetitive and transposable elements. However, this interpretation will require further validation in future studies with larger and more diverse datasets.

### Benchmarking identifier-based feature extraction performance

**Figure 2:**
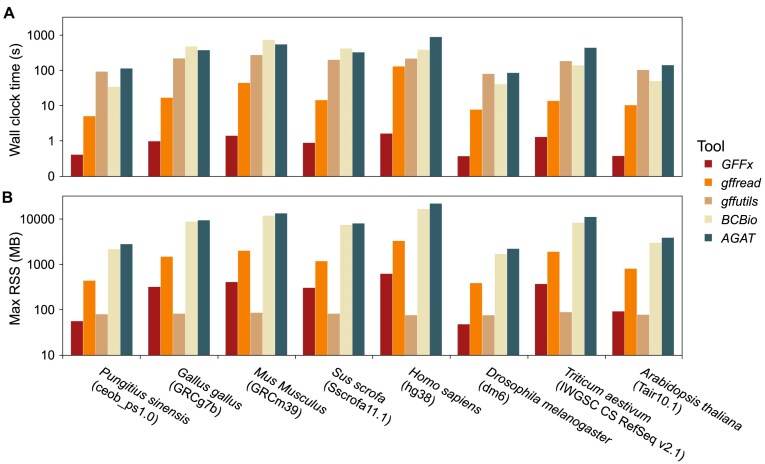
Comparison of identifier-based extraction performance among *GFFx* and other tools. (A) Median wall-clock time (log scale) for extracting 100,000 feature identifiers in different annotation files using *GFFx* (red), *gffread* (orange), *gffutils* (tan), *BCBio* (sand), and *AGAT* (teal). (B) Maximum resident set size (RSS, log scale), a measure of peak memory consumption, for each tool and dataset. Data represent the median of 100 replicate runs.

We benchmarked identifier-based feature extraction performance of *GFFx* against 4 existing tools: *gffread* (v0.12.8) [11], *gffutils* (v0.13) [10], *bcbio-gff* (v0.7.1) [12], and *AGAT* (v1.4.1) [13]. These benchmarks used the same 8 annotation GFF files as above, with 100 replicates per file. In each replicate, we randomly sampled 100,000 feature identifiers once and applied the same subset consistently across all tools to extracted the corresponding entries. *GFFx* achieved median runtimes ranging from 0.37 to 1.62 seconds (Fig. 2A; Supplementary Table S4), corresponding to 10.54- to 80.27-fold speedups over the second fastest tool, *gffread*. Besides, *GFFx* required less memory than other tools, except *gffutils* (Fig. 2B; Supplementary Table S4). Overall, *GFFx* achieves substantial speedups, with the speed increasing proportionally with the size and complexity of the annotation files, without incurring additional memory overhead. As genome assemblies become larger and the annotations grow more detailed, *GFFx* will continue to outpace other tools by an ever-widening margin.

### Benchmarking region-based feature retrieval performance

**Figure 3:**
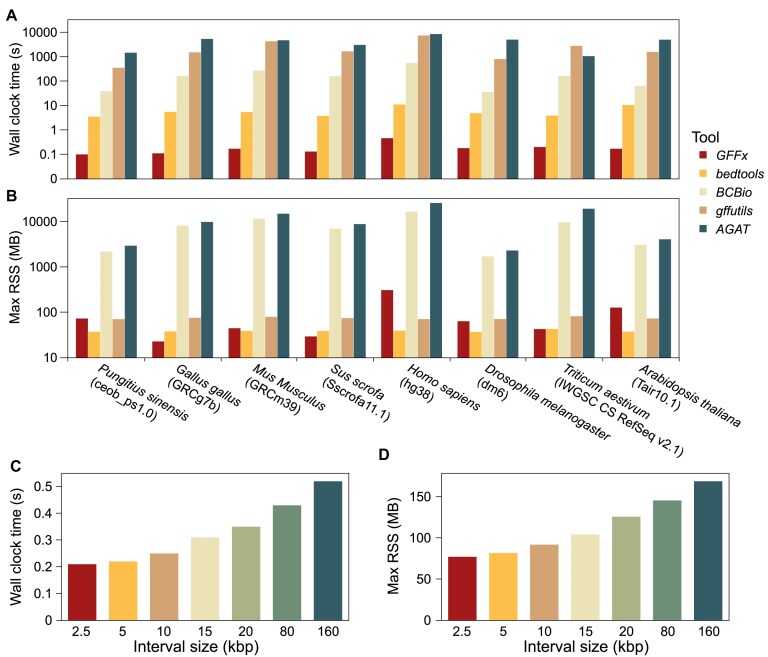
Comparison of region-based feature retrieval performance among *GFFx* and other tools. (A) Median wall-clock time (log scale) for extracting 100,000 random 20-kbp intervals in different genome annotation files using *GFFx* (red), *bedtools* (amber), *gffutils* (tan), *BCBio* (sand), and *AGAT* (teal). (B) Maximum resident set size (RSS, log scale), a measure of peak memory consumption, for each tool and dataset. (C) Median wall-clock time for extracting 100,000 random intervals with sizes ranging from 2.5 to 160 kbp. (D) RSS for extracting 100,000 random intervals with sizes ranging from 2.5 to 160 kbp. Data represent the median of 100 replicate runs.

Subsequently, we compared region-based retrieval performance of *GFFx* against 4 tools—*gffutils, bcbio-gff, AGAT*, and *bedtools* (v2.31.1) [14]—substituting *bedtools* for *gffread* because *gffread* only handles single user-specified regions and does not accept BED files. Using the same 8 annotation GFF files with 100 replicates each, we generated BED4-format interval files containing 100,000 randomly sampled 20-kbp bins per replicate using the random command from *bedtools*. The resulting interval sets were used consistently across all tools within each replicate. Among all tools, *GFFx* delivered the fastest region-based retrieval, with median runtimes ranging from 0.10 to 0.46 seconds (Fig. 3A; Supplementary Table S5). Excluding *GFFx, bedtools* was the next fastest, requiring 3.52 to 11.04 seconds (19.42- to 61.82-fold slower), while dedicated GFF processors were at least 201-fold slower. This performance gain of *GFFx* derives from its interval-tree algorithm, which reduces time complexity from O(*N*) to O(log *N* + *k*), where *N* represents total number of intervals in a GFF file and *k* represents number of overlapped intervals. Although the memory usage of *GFFx* is not always the lowest (Fig. 3B; Supplementary Table S5), it remains under 130 MB across all tests, ensuring operability on standard personal computers without sacrificing speed.

To comprehensively assess *GFFx*’s performance across diverse genomic contexts, we further conducted similar benchmarks on the hg38 annotation using interval lengths ranging from 2.5 to 160 kbp. Across this spectrum, runtime rose gradually from about 0.2 seconds to just over 0.5 seconds (Fig. 3C; Supplementary Table S6), while memory usage increased from ~77 to ~169 MB (Fig. 3D; Supplementary Table S6). Importantly, both measures followed a clear sublinear, power law–like scaling pattern, in which doubling the interval length resulted in only a modest increase of roughly 15% to 17% in computational cost. This behavior highlights the favorable scalability of *GFFx*, demonstrating that the tool retains high efficiency and robustness even under substantially expanded interval lengths, thereby reinforcing its utility in large-scale and heterogeneous genomic analyses.

### Benchmarking performance of coverage profiling

Quantifying coverage of read mapping is a routine need in genomics and computational biology workflows. Diverse sets of genomic intervals (e.g., capture targets, chromatin immunoprecipitation (ChIP) peaks, assay for transposase-accessible chromatin (ATAC) peaks, transcript exons, variant call regions) must be evaluated for how fully they span annotated features or reference coordinates. At scale, this task is challenging because computing exact breadth and depth over large, highly overlapping interval sets is costly, as naive approaches require quadratic overlap checks or per-base scans. It is also difficult to parallelize since overlaps cross partition boundaries and demand global reconciliation. Existing utilities such as *bedtools* provides mature functionality but can become runtime and memory bottlenecks on whole-genome workloads. To address this, *GFFx* introduces 2 dedicated subcommands: *coverage* (for coverage breadth) and *depth* (for coverage depth). By partitioning the genome into indexed slices and combining memory-mapped I/O with interval merging and 2-pointer scans, *GFFx* avoids quadratic checks and enables parallel, memory-bounded computations across independent regions.

We evaluated performance using 2 high-throughput sequencing datasets from *A. thaliana* (NCBI SRA experiment SRX30363821) and *H. sapiens*(NCBI SRA experiment SRX30241060), containing 13.20 million and 40.90 million reads, respectively. For each species, we generated both coordinate-sorted and unsorted BAM files and compared the runtime and memory usage of *GFFx* and *bedtools*. On sorted inputs, *GFFx* ran faster than *bedtools* by 11.58 times in *Arabidopsis* and 14.04 times in *H. sapiens* for breadth and by 10.83 times and 6.93 times for depth (Supplementary Fig. S2a; Supplementary Table S7). With unsorted BAM files, the breadth advantage remained substantial at 8.11 times and 10.15 times in *Arabidopsis* and *H. sapiens*, whereas the depth speedup was more modest at 2.41 times and 1.11 times (Supplementary Fig. S2c; Supplementary Table S7). In all experiments, *GFFx* also required less memory, using as little as one-twentieth of the resident set size observed for *bedtools* (Supplementary Fig. S2b, d; Supplementary Table S7).

## Discussion

Here, we present *GFFx*, a Rust-based, modular, and high-performance toolkit for efficient processing and querying of ultra-large GFF3 genome annotation files. It addresses key limitations of existing tools through a compact, model-aware indexing system and by leveraging Rust’s strengths in speed, memory safety, and multithreaded execution. Many widely used tools suffer from performance bottlenecks when processing large-scale annotations. For example, *gffutils* depends on relational databases, leading to long indexing times and high disk usage; *AGAT* and *bcbio-gff* offer broad functionality but are not optimized for fast querying; *bedtools* supports region-based queries but lacks model awareness; and *gffread* performs well only on small datasets and lacks parallel support.

Region-based queries in *GFFx* are powered by an in-memory interval tree index. Interval trees are a well-established data structure for efficiently storing and querying 1-dimensional intervals that vary widely in length and often overlap or nest, making them an ideal fit for genome annotation data [15]. In an interval tree, each node represents a feature interval and tracks the maximum endpoint of its subtree. This pruning mechanism skips entire subtrees, whose intervals lie outside the query region, avoiding full-file scans and enabling sublinear query times. Once features are identified, *GFFx* uses the *.gof* index, which maps feature IDs to byte offsets in the original GFF file to retrieve annotation blocks directly, resulting in rapid end-to-end extraction even on large, complex datasets.

Benchmark results show that *GFFx* significantly outperforms existing tools in both feature extraction and coverage profiling, offering large speedups while maintaining modest memory usage and strong parallel scalability. As genome annotations continue to grow in complexity and size, *GFFx* offers a practical and extensible foundation for future bioinformatics workflows.

While robust for standard GFF3 files, the current implementation assumes well-formed input and does not yet support GTF or legacy GFF2 formats. Enhancing compatibility and fault tolerance—particularly for nonstandard annotations—remains an important area for development. Planned extensions include support for additional formats, distributed computing integration, and interactive search for large-scale databases. *GFFx* is distributed as a statically compiled binary for Linux, macOS, and Windows. It can also be used as a Rust library, allowing integration into custom pipelines and tools. Its modular architecture and clean API offer fine-grained access to core functions, making *GFFx* both performant and programmable. Full documentation is available at docs.rs/GFFx, and the GitHub repository includes user manuals, benchmarks, input data, and source code for complete reproducibility.

## Methods

### Architectural design of indexing system underpins *GFFx* performance

*GFFx* was developed as a modular and high-performance command-line toolkit for processing large GFF files. Its efficiency is supported by a carefully engineered indexing system (Fig. 1). At the core of *GFFx* are 2 index files shared across all subcommands: *.prt* and *.gof*. The *.prt* index encodes the hierarchical relationships among annotated features and delineates annotation blocks as minimal, biologically coherent units, such as complete gene models or transcript structures. The *.gof* index maps each annotation block to its corresponding byte-offset range in the original GFF file, enabling direct memory-mapped access to specific regions without requiring full-file scanning or decompression. Together, these 2 indices provide the structural and positional backbone of *GFFx*, allowing fast and model-aware access to genome annotations with minimal I/O overhead. To minimize redundancy and reduce index file size, both *.prt* and *.gof* use numeric feature identifiers assigned in order of appearance. The original string-form feature IDs are stored separately in the *.fts* file.

In addition to the core indices, *GFFx* generates several auxiliary index files that support specific subcommands. The extract subcommand retrieves the full annotation block associated with a given feature and requires only the *.fts* index, which records all feature identifiers in order, together with the *.prt* and *.gof* files. For attribute-based queries, the *.atn* file stores all user-specified string-form identifiers found in the attribute field of the GFF file (such as “gene,” “Name,” or “symbol”), while the *.a2f* file maps each attribute value to its corresponding numeric feature ID. These 2 files are used by the search subcommand, which enables both exact and fuzzy attribute queries. The intersect subcommand uses an interval tree scheme. *GFFx* builds a *.rit* file containing all interval tree nodes laid out sequentially and a companion *.rix* file that records offsets in *.rit* for each chromosome or scaffold, so that only the relevant subtree is loaded on demand. This reduces region-query time complexity from O(*N*) to O(log *N*), greatly speeding up lookups in large genomes. All indices are written in compact binary format and accessed on demand by each subcommand to minimize storage footprint and loading time.

### Efficient runtime strategies for feature extraction and coverage profiling

To achieve high-throughput querying from ultra-large GFF3 files, *GFFx* incorporates several performance-oriented design strategies beyond its indexing system. All subcommands operate directly on memory-mapped representations of the original GFF file using the memmap2 library. This eliminates the need for repeated I/O or line-by-line parsing by allowing byte-range access to annotation blocks through read-only mappings. Extracted regions or feature models are located via index lookups and retrieved efficiently by copying their byte slices directly from the memory-mapped buffer. To minimize redundant computation, *GFFx* leverages reference-counted shared memory to ensure that index structures such as *.gof* and .*rit* are loaded only once and reused across all operations. Output blocks are streamed directly to disk, avoiding large memory buffers, and the software assumes well-formed GFF3 input to reduce validation overhead.

To ensure high-performance region-based feature extraction and coverage profiling, *GFFx* leverages several optimizations provided by the Rust ecosystem, such as the use of “FxHashMap” for low-overhead hash-based mappings and “lexical_core” for converting ASCII byte sequences into integer coordinates with minimal latency. Additionally, input regions are pre-bucketed by chromosome and sorted by the start coordinates, ensuring each interval tree to be queried only with relevant regions, thereby reducing unnecessary computation and improving cache locality.

## Availability of Source Code and Requirements

Project name: GFFx

Project homepage: https://github.com/Baohua-Chen/GFFx

Operating system(s): Linux

Programming language: Rust

License: Apache-2.0 license

RRID:SCR_027445

biotools: gffx

## Supplementary Material

giaf124_Supplemental_Files

giaf124_Authors_Response_To_Reviewer_Comments_Orginal_Submission

giaf124_GIGA-D-25-00320_Original_Submission

giaf124_GIGA-D-25-00320_Revision_1

giaf124_Reviewer_1_Report_Original_Submission Xingtan Zhang -- 8/24/2025

giaf124_Reviewer_1_Report_Revision_1 Xingtan Zhang -- 9/29/2025

giaf124_Reviewer_2_Report_Original_Submission Andrew Su -- 9/8/2025

## Data Availability

The source code and user manual of *GFFx* are also archived at Zenodo [16]. Benchmarking scripts and original results are provided at GitHub [17] and Zenodo [16].

## Additional Files

**Supplementary Fig. S1**. Comparison of preprocessing performance between *GFFx* and *gfftuils*. (a) Median wall-clock time (log scale) on different datasets using *GFFx* (red) and *gffutils* (brown). (b) Maximum resident set size (RSS, log scale), a measure of peak memory consumption, for each tool and dataset. (c) Median wall-clock time on hg38 (*Homo sapiens*) downsampled datasets (10%–100%). (d) Maximum resident set size (RSS) on hg38 downsampled datasets (10%–100%).

**Supplementary Fig. S2**. Comparison of coverage profiling performance between *GFFx* and *bedtools*. (a) Median wall-clock time (log scale) for quantifying coverage breadth over Tair10.1 (*Arabidopsis thaliana*) and hg38 (*Homo sapiens*) genome annotations. (b) Maximum resident set size (RSS, log scale) for quantifying breadth over genome annotations. (c) Median wall-clock time (log scale) for quantifying coverage depth over Tair10.1 and hg38 genome annotations. (d) Maximum resident set size (RSS, log scale) for quantifying depth over genome annotations.

**Supplementary Table S1**. File sizes and feature counts of GFF3 annotation datasets used in benchmarking.

**Supplementary Table S2**. Sizes of GFFx index files and gffutils database files from different genome annotation datasets.

**Supplementary Table S3**. Sizes of GFFx index files and gffutils database files from downsampled hg38 GFF files.

**Supplementary Table S4**. Benchmarking runtime and memory usage of ID-based feature extraction across five GFF tools.

**Supplementary Table S5**. Benchmarking runtime and memory usage of region-based feature extraction across 5 GFF tools.

**Supplementary Table S6**. Benchmarking region-based feature extraction performance of *GFFx* using intervals with different sizes.

**Supplementary Table S7**. Benchmarking runtime and memory usage of coverage profiling.

## Abbreviations

GFF: General Feature Format; I/O: input/output; lncRNA: long noncoding RNA; RSS: resident set size.
