# Supplementary material for: GFFx: A Rust-based suite of utilities for ultra-fast genomic feature extraction
Source: Gigascience. 2025 Oct 23;14:giaf124. doi: 10.1093/gigascience/giaf124 (PMC12548526; doi:10.1093/gigascience/giaf124)
Supplement: giaf124_Supplemental_Files [file giaf124_supplemental_files.zip › Supplementary figures.docx]

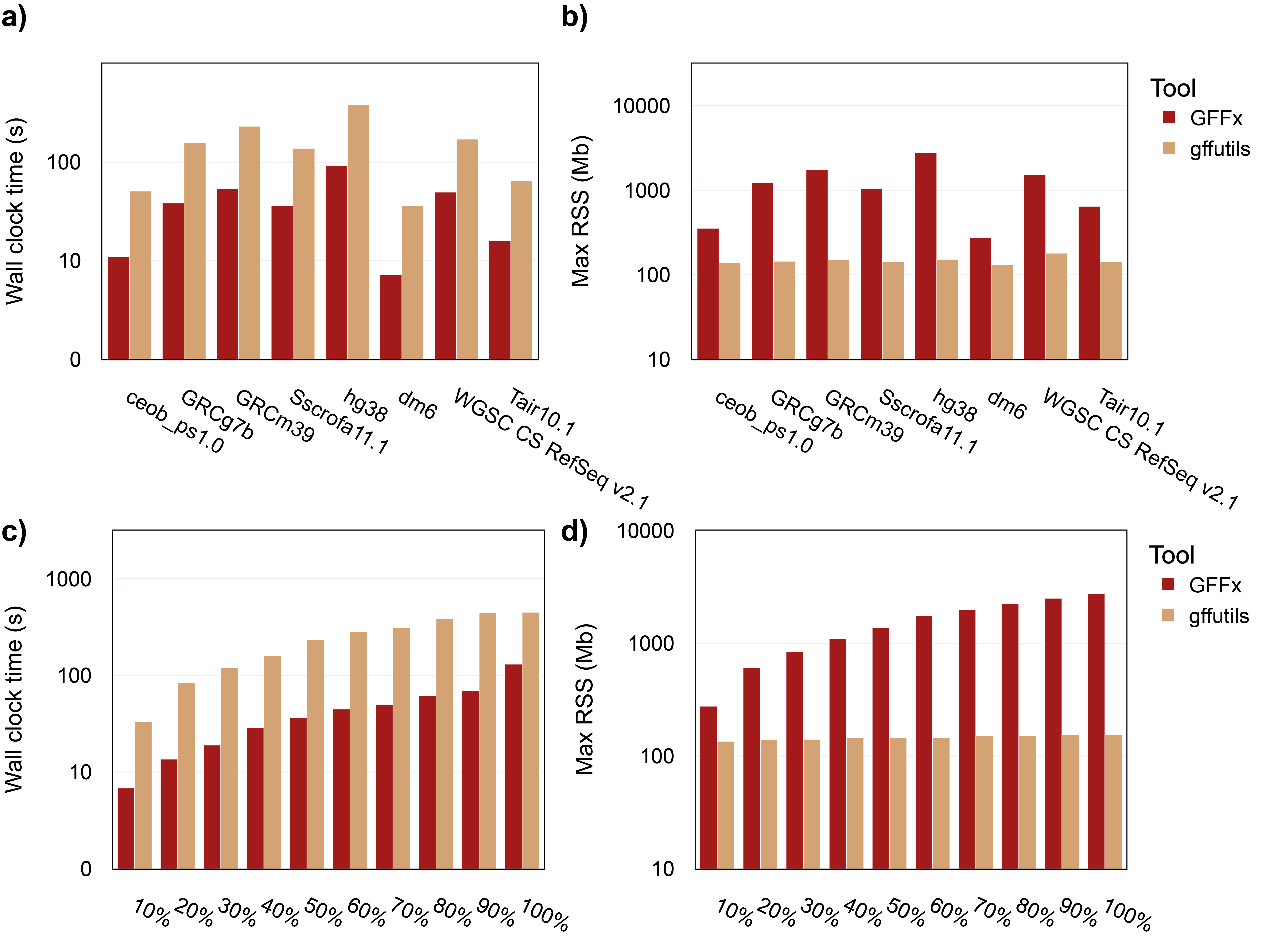


**Figure S1. Comparison of preprocessing performance between *GFFx* and *gfftuils*. (a)** Median wall-clock time (log scale) on different datasets using *GFFx* (red) and *gffutils* (brown). (**b**) Maximum resident set size (RSS, log scale), a measure of peak memory consumption, for each tool and dataset. **(c)** Median wall-clock time on hg38 (*Homo sapiens*) downsampled datasets (10%–100%). **(d)** Maximum resident set size (RSS) on hg38 downsampled datasets (10%–100%).


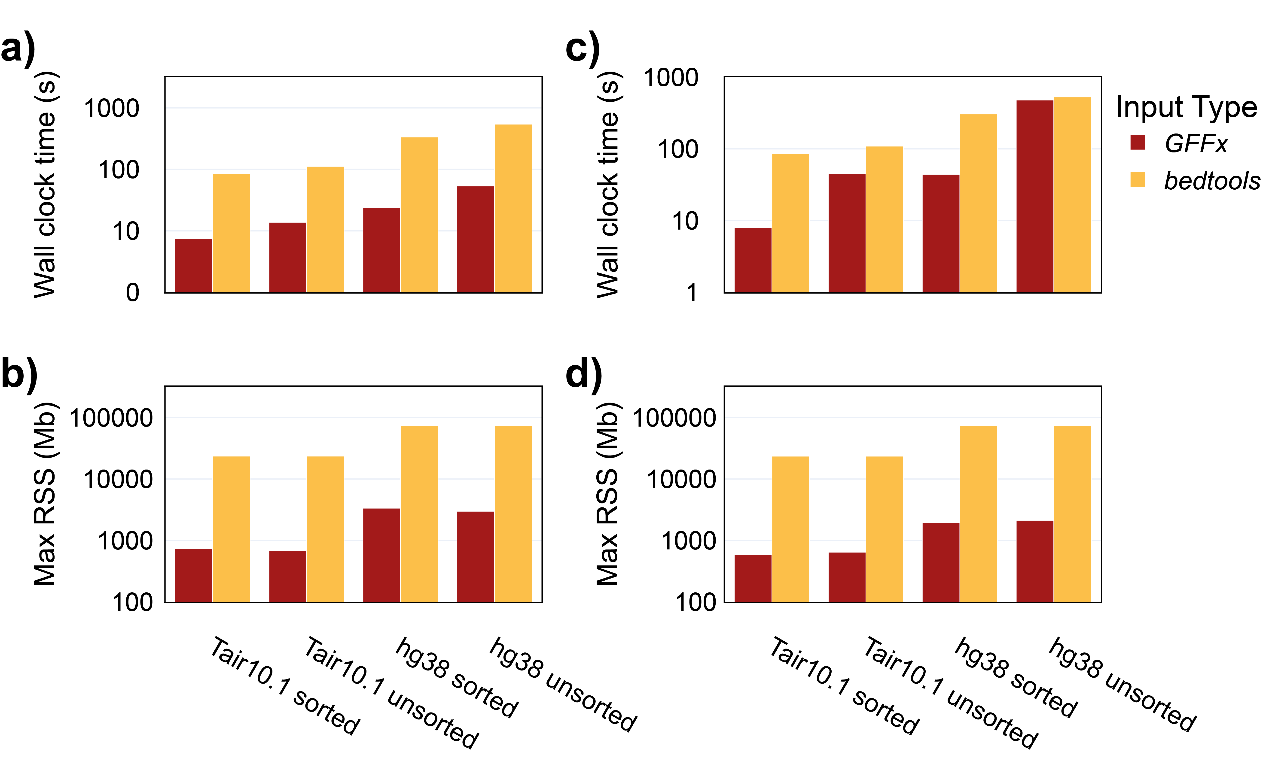


**Figure S2. Comparison of coverage profiling performance between *GFFx* and *bedtools*. (a)** Median wall-clock time (log scale) for quantifying coverage breadth over Tair10.1 (*Arabidopsis thaliana*) and hg38 (*Homo sapiens*) genome annotations. **(b)** Maximum resident set size (RSS, log scale) for quantifying breadth over genome annotations. **(c)** Median wall-clock time (log scale) for quantifying coverage depth over Tair10.1 and hg38 genome annotations. **(d)** Maximum resident set size (RSS, log scale) for quantifying depth over genome annotations.
