# Supplementary material for: GFFx: A Rust-based suite of utilities for ultra-fast genomic feature extraction
Source: Gigascience. 2025 Oct 23;14:giaf124. doi: 10.1093/gigascience/giaf124 (PMC12548526; doi:10.1093/gigascience/giaf124)
Supplement: giaf124_GIGA-D-25-00320_Revision_1 [file giaf124_giga-d-25-00320_revision_1.pdf]

GigaScience

GFFx: A Rust-based suite of utilities for ultra-fast genomic feature extraction

--Manuscript Draft--

**Manuscript Number:**	GIGA-D-25-00320R1
**Full Title:**	GFFx: A Rust-based suite of utilities for ultra-fast genomic feature extraction
**Article Type:**	Technical Note
**Funding Information:**	Young Scientists Fund of the National Natural Science Foundation of China (32300490) — Dr. Dongya Wu<br>National Key R&D Program of China (2025YFC3410304) — Dr. Dongya Wu
**Abstract:**	Genome annotations are becoming increasingly comprehensive due to the discovery of diverse regulatory elements and transcript variants. However, this improvement in annotation resolution poses major challenges for efficient querying, especially across large genomes and pangenomes. Existing tools often exhibit performance bottlenecks when handling large-scale genome annotation files, particularly for region-based queries and hierarchical model extraction. Here, we present GFFx, a Rust-based toolkit for ultra-fast and scalable genome annotation access. GFFx introduces a compact, model-aware indexing system inspired by binning strategies and leverages Rust's strengths in execution speed, memory safety, and multithreading. It supports feature extraction and coverage profiling with significant improvements in runtime and scalability over existing tools. Distributed via Cargo, GFFx provides a cross-platform command-line interface and a reusable library with a clean API, enabling seamless integration into custom pipelines. Benchmark results demonstrate that GFFx offers substantial speedups and makes a practical, extensible solution for genome annotation workflows.
**Corresponding Author:**	Dongya Wu<br>Zhejiang University<br>Hangzhou, CHINA
**Corresponding Author Secondary Information:**	
**Corresponding Author's Institution:**	Zhejiang University
**Corresponding Author's Secondary Institution:**	
**First Author:**	Baohua Chen, Ph. D.
**First Author Secondary Information:**	
**Order of Authors:**	Baohua Chen, Ph. D.
	Dongya Wu
	Guojie Zhang
**Order of Authors Secondary Information:**	
**Response to Reviewers:**	Dear Editors and Reviewers,<br>On behalf of our co-authors, we are pleased to submit a revised version of our manuscript entitled "GFFx: A Rust-Based suite of utilities for ultra-fast genomic feature extraction" for consideration for publication in GigaScience.<br>We appreciate the time and effort that the editors and the reviewers dedicated to providing feedback on our manuscript and are grateful for the insightful comments on and valuable improvements to our paper. We have incorporated all the suggestions made by the reviewers. Those changes are highlighted within the manuscript. Please see attached point-by-point responses to the reviewers' comments and concerns.Additionally, we have registered the new software GFFx in both bio.tools and SciCrunch.org and have got an RRID of SCR_027445 and a biotoolsID of "biotools:gffx".<br>We believe the revisions have substantially improved the manuscript, and we hope it

will now be suitable for publication in GigaScience. We sincerely thank you again for your consideration.

Sincerely,

DongyaWu & Guojie Zhang
Center of Evolutionary & Organismal Biology, Zhejiang University School of Medicine, Hangzhou, 310058, China

Point-by-point response (Manuscript GIGA-D-25-00320)

Reviewer #1:
The overall research appears comprehensive; however, further attention to the tool's capabilities and methodological rigor would strengthen its validity and broader applicability.
Response:
We thank the reviewer for the constructive feedback. We have carefully considered the comments regarding the tool’s capabilities and methodological rigor, and have revised the manuscript accordingly.

1. In the "Performance benchmark in annotation indexing" section, the authors utilized genome annotations from four species (Homo sapiens hg38, Pungitius sinensis ceob_ps_1.0, Drosophila melanogaster dm6, and Arabidopsis thaliana tair10.1) as representatives for benchmarking and subsequent analyses. Nevertheless, a robust GFF processing suite should ideally demonstrate reliability across a broader spectrum of genome types, irrespective of their frequency of use. To enhance the generalizability of GFFx and cater to a wider user base, it is recommended that additional genomes—such as those of Triticum aestivum, Mus musculus, and Sus scrofa—be included in the benchmarks. This would better validate the tool's robustness across species with varying genome complexities.
Response:
Thanks. We fully agree with this suggestion. Accordingly, we have supplemented the benchmarks with analyses based on another four representative species: common wheat (Triticum aestivum, IWGSC CS refseq v2.1), house mouse (Mus musculus, GRCm39), wild boar (Sus scrofa, Sscrofa11.1), and chicken (Gallus gallus, GRCg7b). The results consistently demonstrate the strong performance advantages of GFFx across these additional species. The corresponding results have been added to the revision.

2. While the 20-kb interval length used in the region-based retrieval benchmarks is biologically relevant, corresponding to typical gene sizes, it does not fully capture the diversity of genomic query scenarios. To comprehensively assess GFFx's performance across diverse genomic contexts, it is suggested that supplementary benchmarks be conducted using interval lengths of 10 kb and 100 kb. This would help validate the tool's robustness across varying interval scales, which is critical for its practical utility in diverse research workflows.
Response:
We thank the reviewer for this valuable suggestion and fully agree with the point raised. To address it, we supplemented the benchmarks by using intervals ranging from 2.5 kbp to 160 kbp. The results indicate that both runtime and memory usage of GFFx do increase with larger interval sizes, but the increase is very modest. Specifically, for each doubling of interval size, runtime and memory usage increase by approximately 15-17%. These results have been incorporated into the revised manuscript.

Fig. 3c&3d Median wall-clock time and RSS for extracting 100,000 random intervals with sizes ranging from 2.5 to 160 kbp. Data represent the median of 100 replicate runs. Tools are ordered left-to-right by increasing median wall-clock time on hg38.

3. To further broaden the software's applicability, it is recommended to incorporate an additional functionality that enables the extraction of the number of reads covering specific intervals from BAM files based on positional information derived from GFF3 files, thereby facilitating the calculation of sequencing depth. This feature would be analogous to the functionality provided by `bedtools coverage`, enhancing GFFx's utility in integrating genome annotation data with sequencing read coverage analyses.

Powered by Editorial Manager® and ProduXion Manager® from Aries Systems Corporation

Response:
We thank the reviewer for this insightful comment, which is fully aligned with our own development plan. In fact, the implementation of this functionality had already been initiated as part of the ongoing development of the next version, and during the current manuscript revision we incorporated a preliminary coverage profiling module into GFFx and added a comparison with the widely used tool bedtools. Although the module has not yet undergone extensive optimization, the current implementation already demonstrates clear advantages over bedtools coverage. In particular, when using sorted BAM files as input, the new coverage subcommands for breadth and depth calculation are 6.93- to 14.04-fold faster than bedtools, while also consuming substantially less memory.

Fig S2. Comparison of coverage profiling performance between GFFx and bedtools. (a) Median wall-clock time (log scale) for quantifying coverage breadth over Tair10.1 (Arabidopsis thaliana) and hg38 (Homo sapiens) genome annotations. (b) Maximum resident set size (RSS, log scale) for quantifying breadth over genome annotations. (c) Median wall-clock time (log scale) for quantifying coverage depth over Tair10.1 and hg38 genome annotations. (d) Maximum resident set size (RSS, log scale) for quantifying depth over genome annotations.

Reviewer #2:
This paper describes GFFx, a new fast and efficient toolkit for working with GFF files. The tool describes a notable advance over current state of the art, and the manuscript overall is well-written. I have only the following minor suggestions for consideration:
Response:
Thanks for the positive comments of our work. We have addressed all suggestions as detailed below.

* In figure S1 and the corresponding discussion, the authors test GFFx on 4 different GFF annotation databases of differing sizes, and differences between the performance is attributed solely to the different dataset sizes. The authors should consider subsetting the largest annotation database (hg38) to more smoothly track how performance and memory use vary with annotation database size, and to confirm there are no organism-specific effects that could underlie the observed differences.
Response:
We thank the reviewer for this constructive suggestion. Following the recommendation, we down-sampled the largest annotation database (hg38) to multiple sizes and repeated the benchmarks. The new results show smooth scaling of runtime and memory usage with dataset size, and within hg38 the relative advantage of GFFx over gffutils remained stable across all dataset sizes. By contrast, the variation observed in the cross-organism benchmarks is more likely attributable to differences in annotation complexity rather than dataset size alone. These additional analyses strengthen the conclusion that within a single organism performance is primarily driven by dataset size, while highlighting that the differences across species may reflect more complex biological and annotation-related factors. We note that further studies using a broader range of genomes and annotation styles will be needed to fully disentangle these influences.

Fig. S1 Comparison of preprocessing performance between GFFx and gfftuils. (a) Median wall-clock time (log scale) on different datasets using GFFx (red) and gffutils (brown). (b) Maximum resident set size (RSS, log scale), a measure of peak memory consumption, for each tool and dataset. (c) Median wall-clock time on hg38 (Homo sapiens) down-sampled datasets (10%–100%). (d) Maximum resident set size (RSS) on hg38 down-sampled datasets (10%–100%).

* The authors should consider changing the line charts in figures 2 and 3 to bar charts — I think the line implies a linear relationship between the tools along the x-axis that is not intended.
Response:
We appreciate this helpful suggestion. We have replaced the line charts in Figures 2 and 3 with bar charts, which more appropriately represent the comparison without implying a linear relationship along the x-axis.

Powered by Editorial Manager® and ProduXion Manager® from Aries Systems Corporation

	Fig. 2<br><br>Fig. 3<br><br>* For the purposes of benchmarking, the authors used random sampling to extract subsets of the benchmark datasets (e.g., lines 85 and 107). The authors should confirm that the exact same subsets were used when running each tool.<br>Response:<br>We confirm that for each replicate, the random subsets of feature identifiers or genomic intervals were generated once and applied consistently across all tools, ensuring that the benchmarking results are directly comparable. We have revised the relevant words to clarify this point.<br><br>* In addition to depositing the code and benchmarks on Github, the authors should also deposit snapshots in an archival data repository (like Zenodo).<br>Response:<br>We have deposited snapshots of the code and benchmarking data in Zenodo (10.5281/zenodo.17143647) in addition to GitHub, and the corresponding links have been added to the Data Availability section.
**Additional Information:**	
**Question**	**Response**
Are you submitting this manuscript to a special series or article collection?	No
**Experimental design and statistics**<br><br>Full details of the experimental design and statistical methods used should be given in the Methods section, as detailed in our Minimum Standards Reporting Checklist. Information essential to interpreting the data presented should be made available in the figure legends.<br><br>Have you included all the information requested in your manuscript?	Yes
**Resources**<br><br>A description of all resources used, including antibodies, cell lines, animals and software tools, with enough information to allow them to be uniquely identified, should be included in the Methods section. Authors are strongly encouraged to cite Research Resource Identifiers (RRIDs) for antibodies, model organisms and tools, where possible.<br><br>Have you included the information	Yes

Powered by Editorial Manager® and ProduXion Manager® from Aries Systems Corporation

requested as detailed in our Minimum Standards Reporting Checklist?	
**Availability of data and materials**<br><br>All datasets and code on which the conclusions of the paper rely must be either included in your submission or deposited in publicly available repositories (where available and ethically appropriate), referencing such data using a unique identifier in the references and in the "Availability of Data and Materials" section of your manuscript.<br><br>Have you have met the above requirement as detailed in our Minimum Standards Reporting Checklist?	Yes
GigaScience has policies and guidelines in place for the use of generative AI-writing tools such as ChatGPT. If you have used such writing tools to assist with writing the manuscript this must be declared and cited in the text. Authors should not list AI-writing tools and other AI-assisted technologies as an author or co-author and should acknowledge that they are fully responsible for text generated or refined by AI-writing tools.<p><br><br>A summary of use (particularly in the introduction or among methods) needs to be included at the end of the paper, and the outputs should also be included as a supplementary file hosted in GigaDB or other open repositories. Please read our guidelines for more information. <p><br><br>By submitting to GigaScience, you are aware of the journal's AI-writing tools policy, and if you have declared use of such tools below, you have acknowledged this where appropriate in your manuscript and have made a summary of use and outputs available. <p><br><b>AI-assisted writing tools have been used in the preparation of this manuscript?	No

Manuscript

Click here to access/download;Manuscript;GFFx_Giga_MS_Sep 30.docx

GFFx: A Rust-based suite of utilities for ultra-fast genomic feature extraction

Baohua Chen[1,2], Dongya Wu[1,2,*], Guojie Zhang[1,2,*]

[1]School of Basic Medical Sciences, Zhejiang University School of Medicine, Hangzhou 310058, China

[2]Center for Evolutionary & Organismal Biology, Liangzhu Laboratory, Zhejiang University Medical Center, Hangzhou 311121, China

*Corresponding Authors: Dongya Wu, email: wudongya@zju.edu.cn; Guojie Zhang, email: guojiezhang@zju.edu.cn

Baohua Chen [0000-0002-3065-0739]; Dongya Wu [0000-0003-1967-2264]; Guojie Zhang [0000-0001-6860-1521]

Abstract

Genome annotations are becoming increasingly comprehensive due to the discovery of diverse regulatory elements and transcript variants. However, this improvement in annotation resolution poses major challenges for efficient querying, especially across large genomes and pangenomes. Existing tools often exhibit performance bottlenecks when handling large-scale genome annotation files, particularly for region-based queries and hierarchical model extraction. Here, we present *GFFx*, a Rust-based toolkit for ultra-fast and scalable genome annotation access. *GFFx* introduces a compact, model-aware indexing system inspired by binning strategies and leverages Rust's strengths in execution speed, memory safety, and multithreading. It supports feature extraction and coverage profiling with significant improvements in runtime and scalability over existing tools. Distributed via Cargo, *GFFx* provides a cross-platform command-line interface and a reusable library with a clean API, enabling seamless integration into custom pipelines. Benchmark results demonstrate that *GFFx* offers substantial speedups and makes a practical, extensible solution for genome annotation workflows.

Keywords: GFF file; Genome Annotation; Rust Programming; Mapping Coverage

Introduction

With the growing understanding of functional genome regions beyond conventional protein-coding genes, genome annotations are rapidly increasing in both complexity and volume. Large-scale efforts such as ENCODE [1], FANTOM [2], and Roadmap Epigenomics Program [3] have cataloged diverse noncoding elements—including enhancers, promoters, long non-coding RNAs (lncRNAs), and epigenetic marks—highlighting their roles in gene regulation, chromatin

dynamics, and cellular identity. As novel regulatory elements, alternative isoforms, and lineage- or tissue-specific transcripts continue to emerge, annotation datasets are expected to expand further [4]. The accumulation of such multilayered annotations, particularly across large genomes or pangenomes, poses growing challenges for storage, indexing, and efficient querying.

However, existing tools often struggle to process ultra-large annotation files efficiently, particularly for region-based queries, hierarchical model extraction, or parallel execution. A scalable, high-performance solution optimized for such tasks is urgently needed. Rust, a modern systems programming language, offers high execution speed, memory safety, efficient multithreading, and cross-platform portability. These features have led to its increasing adoption in bioinformatics [5], as exemplified by Rust-Bio [6], Bigtools [7], Phylo-rs [8], and fibertools [9].

To address these challenges, we developed *GFFx*, a Rust-based toolkit for fast and scalable access to genome annotation files. *GFFx* supports region-, identity-, and attribute-based queries over ultra-large General Feature Format (GFF) datasets. Designed as both a command-line tool and a reusable library, it can be integrated into larger pipelines and software systems. It also demonstrates Rust's potential in computational biology by providing a robust, extensible foundation for high-performance annotation processing.

Findings

Performance benchmark in annotation indexing

GFFx achieves high-performance efficiency through a modular indexing system anchored by two core indices, .prt and .gof, which capture feature hierarchical relationship and map annotation blocks to their byte-offsets for direct memory access, respectively. Complementary lightweight indices, including *.fts*, *.a2f*, *.atn*, *.sqs*, *rit*, and *.rix*, support subcommand-specific operations like feature extraction, attribute-based searches, and region queries with minimal I/O overhead (**Fig. 1**).

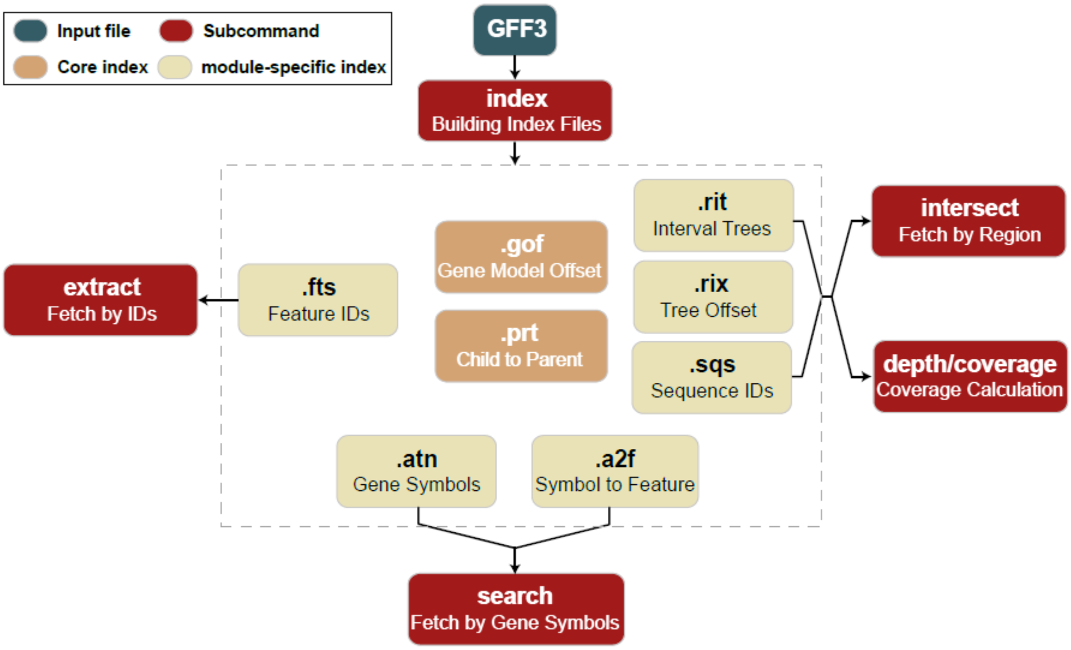

Figure 1. Architecture of the indexing system and subcommand interactions in *GFFx*. All index files are generated in advance from a GFF3 file (cream box) via the index module. While all subcommands (green boxes) have access to the complete set of indices, each subcommand loads only the subset relevant to its specific function. Core indices *.gof* and *.prt* (dark brown boxes) are universally required, whereas module-specific indices, including *.fts*, *.a2f*, *.atn*, *.rit*, *.rix*, and *.sqs* (light brown boxes) are utilized only by specific subcommands as illustrated.

Among commonly used GFF processing tools, only *gffutils* [10] performs preprocessing by converting GFF files into an SQLite database. In contrast, *GFFx* adopts a lightweight index strategy optimized for direct file-based access. To assess the relative efficiency of these two approaches, we compared the runtime required for index construction in *GFFx* versus database creation in *gffutils* (v0.13).

For this evaluation, we selected eight representative GFF3 annotation datasets spanning a broad taxonomic range and varying annotation complexities, with file sizes ranging from 156.86 to 1511.79 MB (**Supplementary Table S1**). The datasets included the vertebrate genomes of *Pungitius sinensis* (ceob_ps_1.0), *Gallus gallus* (GRCg7b), *Mus Musculus* (GRCm39), *Sus scrofa* (Sscrofa11.1) and *Homo sapiens* genome (hg38), as well as the invertebrate genome of *Drosophila melanogaster* (dm6), and two plant genomes, *Triticum aestivum* (IWGSC CS refseq v2.1) and *Arabidopsis thaliana* (Tair10.1). These datasets collectively capture the diversity of genome sizes and annotation scales observed in contemporary genomics. All benchmarks were performed on a dedicated compute node equipped with 2 × Intel(R) Xeon(R) Gold 6448H CPUs (32 cores/64 threads each), 1 TB DDR4 RAM, and dual Micron 7450 MTFDKCB960TFR NVMe SSDs (total capacity 1.92 TB). Despite its relatively complex indexing architecture, *GFFx*

consistently outperformed *gffutils*, achieving speedups of 5.81- to 8.45-fold (**Supplementary Fig. S1a**). This improvement was accompanied by higher memory usage. For the largest dataset hg38, *GFFx* required 2.77 GB of memory, which remains manageable on most modern computing platforms including personal computers (**Supplementary Fig. S1b**). In addition, the sizes of the index files produced by both tools scaled linearly with dataset size, and the indexes generated by *GFFx* were about 2.5 to 4.1 percent of the size of those produced by *gffutils* (**Supplementary Tables S2, 3**), underscoring another key advantage of *GFFx*.

To assess the effect of dataset size within a single organism, we down-sampled hg38 and repeated the benchmarks. Runtime increased with dataset size for both tools, and *GFFx* consistently finished in about one sixth to one seventh of the time required by *gffutils* (**Supplementary Fig. S1c**). Memory usage for *GFFx* increased nearly linearly with dataset size, whereas *gffutils* remained almost constant (**Supplementary Fig. S1d**). Within hg38, these results indicate size-driven scaling with a stable relative advantage of *GFFx*. In contrast, cross-organism comparisons show more variability in the relative speedup, which is more plausibly explained by differences in annotation complexity, such as the density of non-coding RNAs, the prevalence of alternative splicing, and the abundance of repetitive and transposable elements. However, this interpretation will require further validation in future studies with larger and more diverse datasets.

Benchmarking identifier-based feature extraction performance

We benchmarked identifier-based feature extraction performance of *GFFx* against four existing tools: *gffread* (v0.12.8) [11], *gffutils* (v0.13) [10], *bcbio-gff* (v0.7.1) [12] and *AGAT* (v1.4.1) [13]. These benchmarks used the same eight annotation GFF files as above, with 100 replicates per file. In each replicate, we randomly sampled 100,000 feature identifiers once and applied the same subset consistently across all tools to extracted the corresponding entries. *GFFx* achieved median runtimes ranging from 0.37 to 1.62 s (**Fig. 2a**; **Supplementary Table S4**), corresponding to 10.54- to 80.27-fold speedups over the second fastest tool, *gffread*. Besides, *GFFx* required less memory than other tools except *gffutils* (**Fig. 2b**; **Supplementary Table S4**). Overall, *GFFx* achieves substantial speedups, with the speed increasing proportionally with the size and complexity of the annotation files, without incurring additional memory overhead. As genome assemblies become larger and the annotations grow more detailed, *GFFx* will continue to outpace other tools by an ever-widening margin.

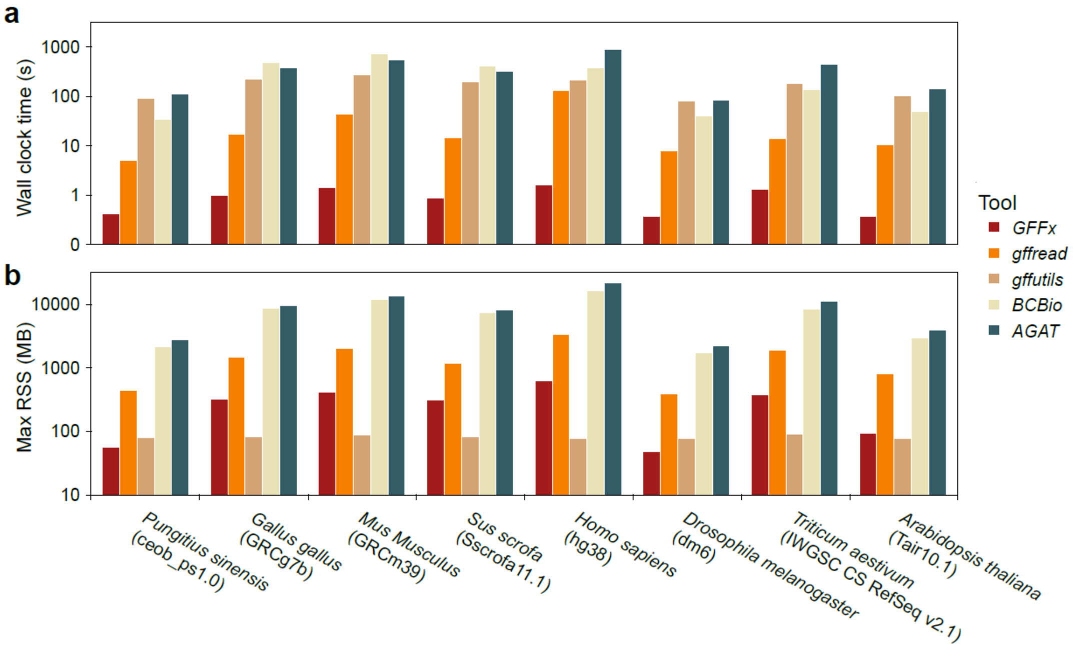

Figure 2. Comparison of identifier-based extraction performance among *GFFx* and other tools. (a) Median wall-clock time (log scale) for extracting 100,000 feature identifiers in different annotation files using *GFFx* (red), *gffread* (orange), *gffutils* (tan), *BCBio* (sand), and *AGAT* (teal). **(b)** Maximum resident set size (RSS, log scale), a measure of peak memory consumption, for each tool and dataset. Data represents the median of 100 replicate runs.

Benchmarking region-based feature retrieval performance

Subsequently, we compared region-based retrieval performance of *GFFx* against four tools—*gffutils*, *bcbio-gff*, *AGAT* and *bedtools* (v2.31.1) [14]—substituting *bedtools* for *gffread* because *gffread* only handles single user-specified regions and does not accept BED files. Using the same eight annotation GFF files with 100 replicates each, we generated BED4-format interval files containing 100,000 randomly sampled 20-kbp bins per replicate using the random command from *bedtools*. The resulting interval sets were used consistently across all tools within each replicate. Among all tools, *GFFx* delivered the fastest region-based retrieval, with median runtimes ranging from 0.10 s to 0.46 s (**Fig. 3a**; **Supplementary Table S5**). Excluding *GFFx*, *bedtools* was the next fastest, requiring 3.52–11.04 s (19.42–61.82-fold slower), while dedicated GFF processors were at least 201-fold slower. This performance gain of *GFFx* derives from its interval-tree algorithm, which reduces time complexity from O(N) to O(log $N + k$), where the N represents total number of intervals in a GFF file and the k represents number of overlapped intervals. Although the memory usage of *GFFx* is not always the lowest (**Fig. 3b**; **Supplementary Table S5**), it remains under 130 MB across all tests, ensuring operability on standard personal computers without sacrificing speed.

To comprehensively assess *GFFx*'s performance across diverse genomic contexts, we further conducted similar benchmarks on the hg38 annotation using interval lengths ranging from 2.5 kbp up to 160 kbp. Across this spectrum, runtime rose gradually from about 0.2 s to just over 0.5 s (**Fig. 3c**; **Supplementary Table S6**), while memory usage increased from ~77 MB to ~169 MB (**Fig. 3d**; **Supplementary Table S6**). Importantly, both measures followed a clear sublinear, power-law–like scaling pattern, in which doubling the interval length resulted in only a modest increase of roughly 15–17% in computational cost. This behavior highlights the favorable scalability of *GFFx*, demonstrating that the tool retains high efficiency and robustness even under substantially expanded interval lengths, thereby reinforcing its utility in large-scale and heterogeneous genomic analyses.

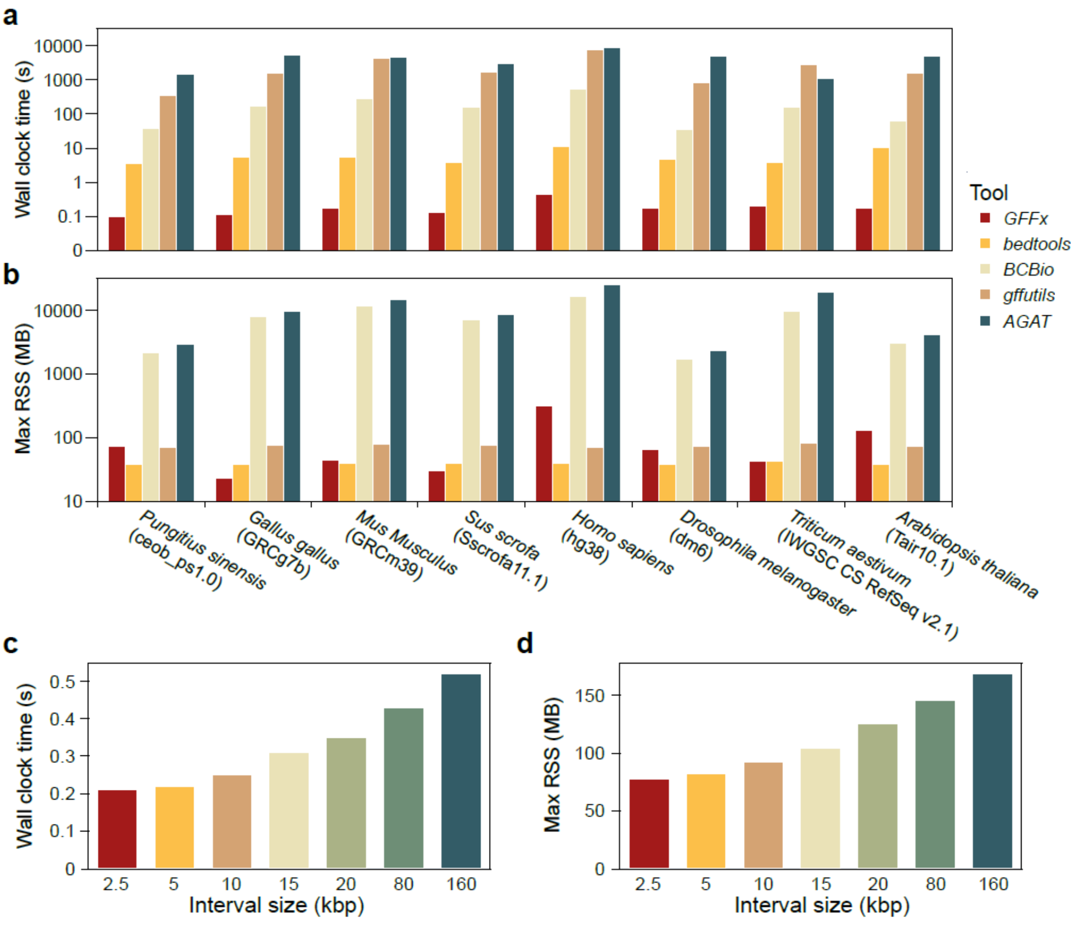

Figure 3. Comparison of region-based feature retrieval performance among *GFFx* and other tools. (a) Median wall-clock time (log scale) for extracting 100,000 random 20-kbp intervals in different genome annotation files using *GFFx* (red), *bedtools* (amber), *gffutils* (tan), *BCBio* (sand), and *AGAT* (teal). **(b)** Maximum resident set size (RSS, log scale), a measure of peak memory consumption, for each tool and dataset. **(c)** Median wall-clock time for extracting 100,000 random intervals with sizes ranging from 2.5 to 160 kbp. **(d)** RSS for extracting 100,000 random intervals with sizes ranging from 2.5 to 160 kbp. Data represent the median of 100 replicate runs.

Benchmarking performance of coverage profiling

Quantifying coverage of read mapping is a routine need in genomics and computational biology workflows. Diverse sets of genomic intervals (e. g. capture targets, ChIP/ATAC peaks, transcript exons, variant call regions) must be evaluated for how fully they span annotated features or reference coordinates. At scale, this task is challenging because computing exact breadth and depth over large, highly overlapping interval sets is costly, as naïve approaches require quadratic overlap checks or per-base scans. It is also difficult to parallelize since overlaps cross partition boundaries and demand global reconciliation. Existing utilities such as *bedtools* provides mature functionality but can become runtime and memory bottlenecks on whole-genome workloads. To address this, *GFFx* introduces two dedicated subcommands: *coverage* (for coverage breadth) and *depth* (for coverage depth). By partitioning the genome into indexed slices and combining memory-mapped I/O with interval merging and two-pointer scans, *GFFx* avoids quadratic checks and enables parallel, memory-bounded computations across independent regions.

We evaluated performance using two high-throughput sequencing datasets from *Arabidopsis thaliana* (NCBI SRA experiment SRX30363821) and human (NCBI SRA experiment SRX30241060), containing 13.20 million and 40.90 million reads, respectively. For each species we generated both coordinate-sorted and unsorted BAM files and compared the runtime and memory usage of *GFFx* and *bedtools*. On sorted inputs *GFFx* ran faster than *bedtools* by 11.58 times in *Arabidopsis* and 14.04 times in human for breadth and by 10.83 times and 6.93 times for depth (**Supplementary Fig. S2a**; **Supplementary Table S7**). With unsorted BAM files the breadth advantage remained substantial at 8.11 times and 10.15 times in *Arabidopsis* and human whereas the depth speedup was more modest at 2.41 times and 1.11 times (**Supplementary Fig. S2c**; **Supplementary Table S7**). In all experiments *GFFx* also required less memory using as little as one twentieth of the resident set size observed for *bedtools* (**Supplementary Fig. S2b, d**; **Supplementary Table S7**).

Discussion

Here, we present *GFFx*, a Rust-based, modular, and high-performance toolkit for efficient processing and querying of ultra-large GFF3 genome annotation files. It addresses key limitations of existing tools through a compact, model-aware indexing system and by leveraging Rust's strengths in speed, memory safety, and multithreaded execution. Many widely used tools suffer from performance bottlenecks when processing large-scale annotations. For example, *gffutils* depends on relational databases, leading to long indexing times and high disk usage; *AGAT* and

bcbio-gff offer broad functionality but are not optimized for fast querying; *bedtools* supports region-based queries but lacks model awareness; and *gffread* performs well only on small datasets and lacks parallel support.

Region-based queries in *GFFx* are powered by an in-memory interval-tree index. Interval trees are a well-established data structure for efficiently storing and querying one-dimensional intervals that vary widely in length and often overlap or nest, making them an ideal fit for genome annotation data [15]. In an interval tree, each node represents a feature interval and tracks the maximum endpoint of its subtree. This pruning mechanism skips entire subtrees whose intervals lie outside the query region, avoiding full-file scans and enabling sublinear query times. Once features are identified, *GFFx* uses the *.gof* index, which maps feature IDs to byte offsets in the original GFF file, to retrieve annotation blocks directly, resulting in rapid end-to-end extraction even on large, complex datasets.

Benchmark results show that *GFFx* significantly outperforms existing tools in both feature extraction and coverage profiling, offering large speedups while maintaining modest memory usage and strong parallel scalability. As genome annotations continue to grow in complexity and size, *GFFx* offers a practical and extensible foundation for future bioinformatics workflows.

While robust for standard GFF3 files, the current implementation assumes well-formed input and does not yet support GTF or legacy GFF2 formats. Enhancing compatibility and fault tolerance—particularly for nonstandard annotations—remains an important area for development. Planned extensions include support for additional formats, distributed computing integration, and interactive search for large-scale databases. *GFFx* is distributed as a statically compiled binary for Linux, macOS, and Windows. It can also be used as a Rust library, allowing integration into custom pipelines and tools. Its modular architecture and clean API offer fine-grained access to core functions, making *GFFx* both performant and programmable. Full documentation is available at docs.rs/GFFx, and the GitHub repository includes user manuals, benchmarks, input data, and source code for complete reproducibility.

Methods

Architectural design of indexing system underpins GFFx performance

GFFx was developed as a modular and high-performance command-line toolkit for processing large GFF files. Its efficiency is supported by a carefully engineered indexing system (**Fig. 1**). At the core of *GFFx* are two index files shared across all subcommands: *.prt* and *.gof*. The *.prt* index encodes the hierarchical relationships among annotated features and delineates annotation blocks as minimal, biologically coherent units, such as complete gene models or transcript structures. The *.gof* index maps each annotation block to its corresponding byte-offset range in the original

GFF file, enabling direct memory-mapped access to specific regions without requiring full-file scanning or decompression. Together, these two indices provide the structural and positional backbone of *GFFx*, allowing fast and model-aware access to genome annotations with minimal input/output overhead. To minimize redundancy and reduce index file size, both *.prt* and *.gof* use numeric feature identifiers assigned in order of appearance. The original string-form feature IDs are stored separately in the *.fts* file.

In addition to the core indices, *GFFx* generates several auxiliary index files that support specific subcommands. The extract subcommand retrieves the full annotation block associated with a given feature and requires only the *.fts* index, which records all feature identifiers in order, together with the *.prt* and *.gof* files. For attribute-based queries, the *.atn* file stores all user-specified string-form identifiers found in the attribute field of the GFF file (such as “gene”, “Name”, or “symbol”), while the *.a2f* file maps each attribute value to its corresponding numeric feature ID. These two files are used by the search subcommand, which enables both exact and fuzzy attribute queries. The intersect subcommand uses an interval tree scheme. *GFFx* builds a *.rit* file containing all interval-tree nodes laid out sequentially and a companion *.rix* file that records offsets in *.rit* for each chromosome or scaffold, so that only the relevant subtree is loaded on demand. This reduces region-query time complexity from O(N) to O(log N), greatly speeding up lookups in large genomes. All indices are written in compact binary format and accessed on demand by each subcommand to minimize storage footprint and loading time.

Efficient Runtime Strategies for Feature Extraction and Coverage Profiling

To achieve high-throughput querying from ultra-large GFF3 files, *GFFx* incorporates several performance-oriented design strategies beyond its indexing system. All subcommands operate directly on memory-mapped representations of the original GFF file using the memmap2 library. This eliminates the need for repeated I/O or line-by-line parsing by allowing byte-range access to annotation blocks through read-only mappings. Extracted regions or feature models are located via index lookups and retrieved efficiently by copying their byte slices directly from the memory-mapped buffer. To minimize redundant computation, *GFFx* leverages reference-counted shared memory to ensure that index structures such as *.gof* and *.rit* are loaded only once and reused across all operations. Output blocks are streamed directly to disk, avoiding large memory buffers, and the software assumes well-formed GFF3 input to reduce validation overhead.

To ensure high-performance region-based feature extraction and coverage profiling, *GFFx* leverages several optimizations provided by the Rust ecosystem, such as the use of “FxHashMap” for low-overhead hash-based mappings and “lexical_core” for converting ASCII byte sequences into integer coordinates with minimal latency. Additionally, input regions are pre-bucketed by

chromosome and sorted by the start coordinates, ensuring each interval tree to be queried only with relevant regions, thereby reducing unnecessary computation and improving cache locality.

Supplementary data

Supplementary data are available online.

Conflict of interest

The authors declare no conflict of interest.

Author contributions

B.C. conceived the project, implemented the method, analyzed the data and wrote the manuscript draft. D.W. discussed the results and revised the manuscript. G.Z. supervised this study.

Availability of Source Code and Requirements

Project name: GFFx

Project homepage: https://github.com/Baohua-Chen/GFFx

Operating system(s): Linux

Programming language: Rust

License: Apache-2.0 license

RRID: SCR_027445

biotools: gffx

Data availability

The source code and user manual of GFFx are also archived at Zenodo [16] Benchmarking scripts and original results are provided at GitHub [17] and Zenodo [16].

Funding

This work was supported by National Key R&D Program of China (2025YFC3410304) and the Young Scientists Fund of the National Natural Science Foundation of China (No. 32300490) to D.W.

References

1. Moore JE, Purcaro MJ, Pratt HE, Epstein CB, Shoresh N, Adrian J, et al.. Expanded encyclopaedias of DNA elements in the human and mouse genomes. *Nature*. Nature Publishing Group; 2020; doi: 10.1038/s41586-020-2493-4.

2. Forrest ARR, Kawaji H, Rehli M, Kenneth Baillie J, de Hoon MJL, Haberle V, et al.. A promoter-level mammalian expression atlas. *Nature*. Nature Publishing Group; 2014; doi: 10.1038/nature13182.

3. Satterlee JS, Chadwick LH, Tyson FL, McAllister K, Beaver J, Birnbaum L, et al.. The NIH Common Fund/Roadmap Epigenomics Program: Successes of a comprehensive consortium. *Science Advances*. American Association for the Advancement of Science; 2019; doi: 10.1126/sciadv.aaw6507.

4. Harrison PW, Amode MR, Austine-Orimoloye O, Azov AG, Barba M, Barnes I, et al.. Ensembl 2024. *Nucleic Acids Research*. 2024; doi: 10.1093/nar/gkad1049.

5. Jeffrey M. Perkel. Why scientists are turning to Rust. *Nature*. 588:1852020;

6. Köster J. Rust-Bio: a fast and safe bioinformatics library. *Bioinformatics*. 2016; doi: 10.1093/bioinformatics/btv573.

7. Huey JD, Abdennur N. Bigtools: a high-performance BigWig and BigBed library in Rust. *Bioinformatics*. 2024; doi: 10.1093/bioinformatics/btae350.

8. Vijendran S, Anderson T, Markin A, Eulenstein O. Phylo-rs: an extensible phylogenetic analysis library in rust. *BMC Bioinformatics*. 2025; doi: 10.1186/s12859-025-06234-w.

9. Jha A, Bohaczuk SC, Mao Y, Ranchalis J, Mallory BJ, Min AT, et al.. DNA-m6A calling and integrated long-read epigenetic and genetic analysis with fibertools. *Genome Res*. 2024; doi: 10.1101/gr.279095.124.

10. Dale R. Gffutils: GFF and GTF file manipulation and intercon-version. Github;

11. Pertea G, Pertea M. GFF Utilities: GffRead and GffCompare. *F1000Res*. 2020; doi: 10.12688/f1000research.23297.2.

12. Chapman B. bcbio-gff. GitHub. v0. 6.4.

13. Dainat J. Another Gtf/Gff Analysis Toolkit (AGAT): Resolve Interoperability Issues and Accomplish More with Your Annotations. *Plant and Animal Genome XXIX Conference (January 8-12, 2022)*. PAG;

14. Quinlan AR, Hall IM. BEDTools: a flexible suite of utilities for comparing genomic features. *Bioinformatics*. 2010; doi: 10.1093/bioinformatics/btq033.

15. Lawrence M, Huber W, Pagès H, Aboyoun P, Carlson M, Gentleman R, et al.. Software for

Computing and Annotating Genomic Ranges. *PLOS Computational Biology*. Public Library of Science; 2013; doi: 10.1371/journal.pcbi.1003118.

16. Chen B, Wu D, Zhang G. GFFx: A Rust-based suite of utilities for ultra-fast genomic feature extraction. In GFFx: A Rust-based suite of utilities for ultra-fast genomic feature extraction. Zenodo. 2025. https://doi.org/10.5281/zenodo.17143647.

17. Chen B. GFFx_benchmarks. Github website. https://github.com/Baohua-Chen/GFFx_benchmarks. Accessed August 20, 2025.

Fig1

Click here to access/download;Figure;Fig1.schema.pdf

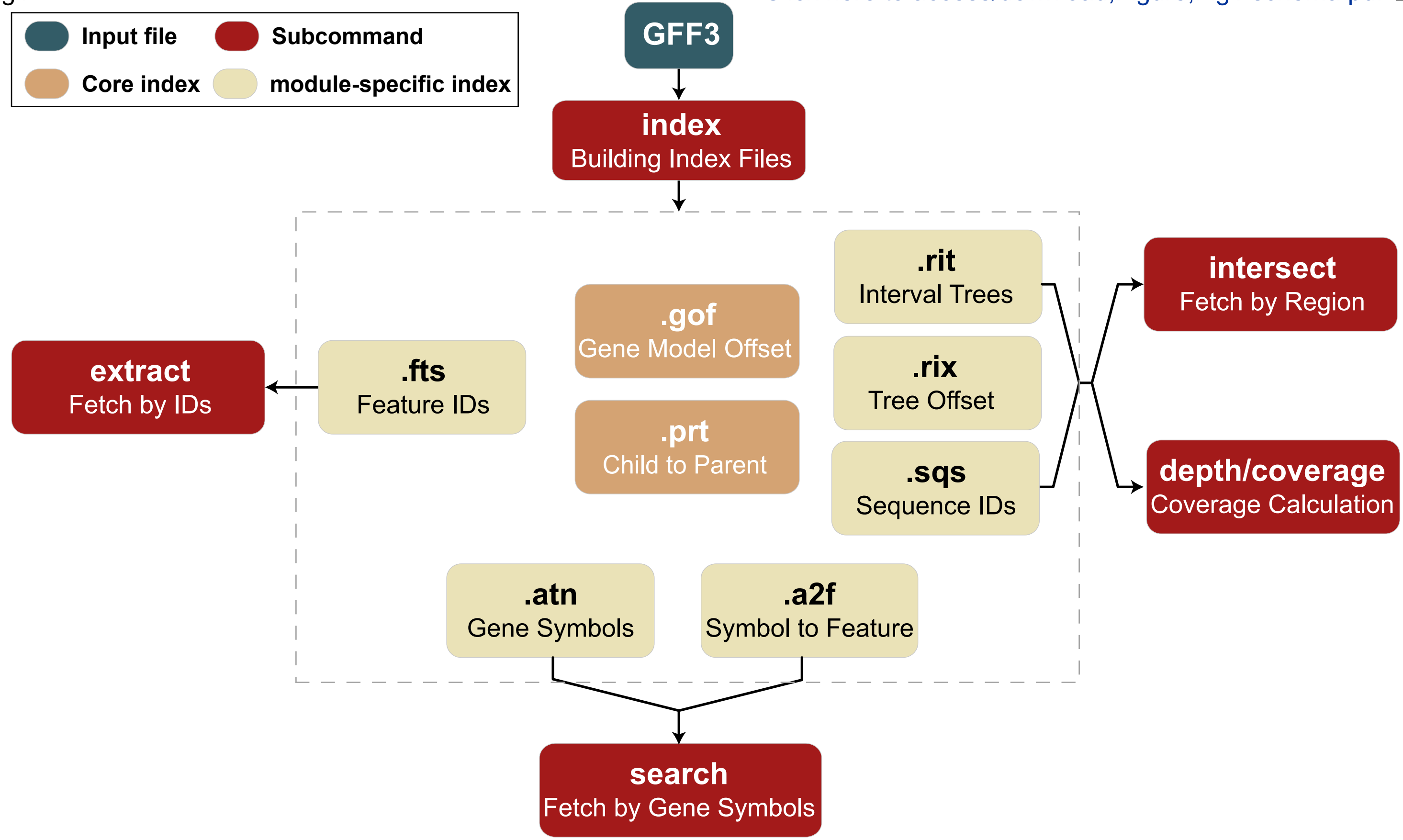

Click here to access/download;Figure;Fig2.extract.pdf

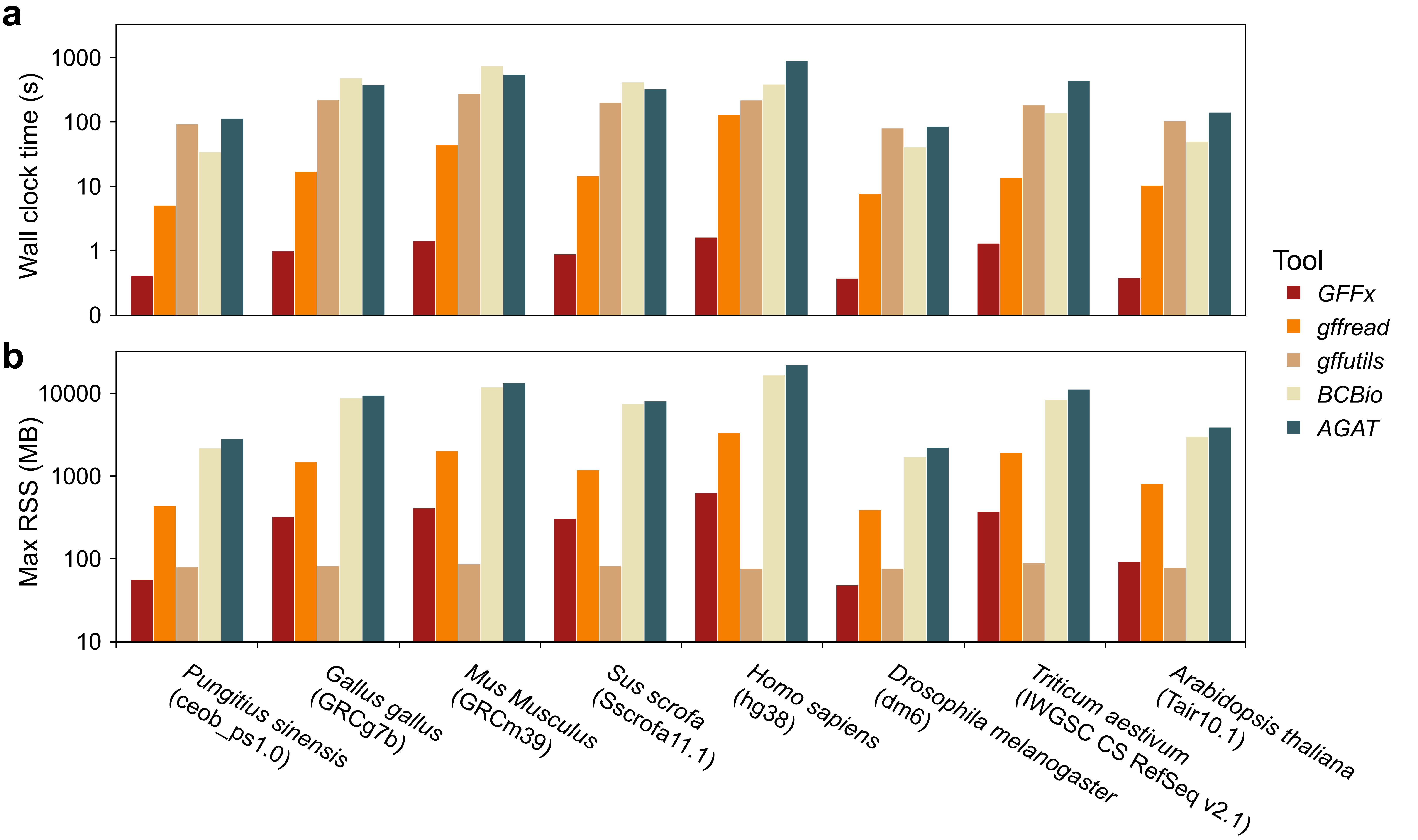

Fig3

Click here to access/download;Figure;Fig3.intersect.pdf

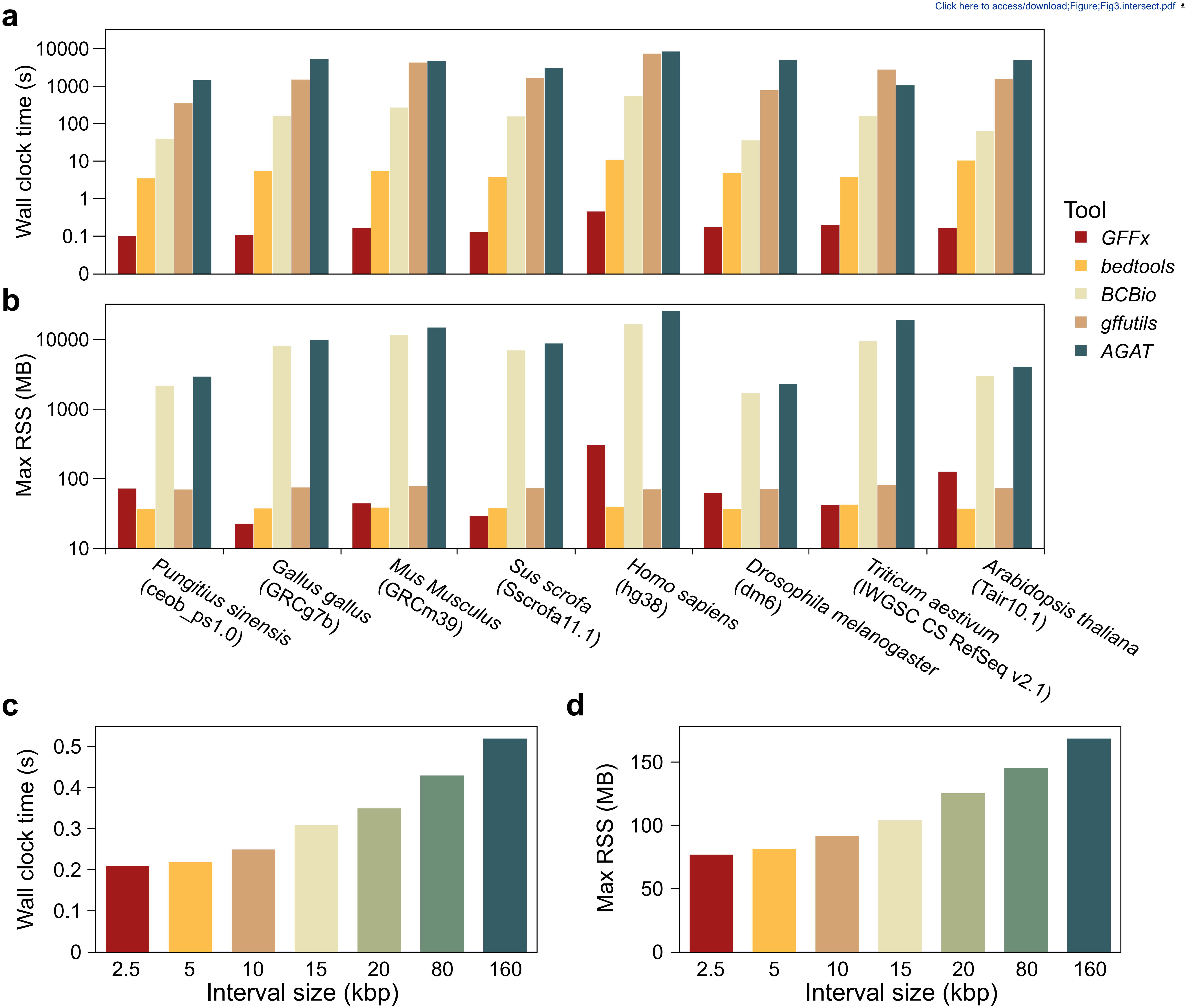

Click here to access/download
Supplementary Material
Supplementary figures.docx

Click here to access/download
Supplementary Material
Supplementary tables.xlsx

Point-by-point response (Manuscript GIGA-D-25-00320)

Reviewer #1:
The overall research appears comprehensive; however, further attention to the tool's capabilities and methodological rigor would strengthen its validity and broader applicability.
Response:
We thank the reviewer for the constructive feedback. We have carefully considered the comments regarding the tool's capabilities and methodological rigor, and have revised the manuscript accordingly.

1. In the "Performance benchmark in annotation indexing" section, the authors utilized genome annotations from four species (*Homo sapiens* hg38, *Pungitius sinensis* ceob_ps_1.0, *Drosophila melanogaster* dm6, and *Arabidopsis thaliana* tair10.1) as representatives for benchmarking and subsequent analyses. Nevertheless, a robust GFF processing suite should ideally demonstrate reliability across a broader spectrum of genome types, irrespective of their frequency of use. To enhance the generalizability of GFFx and cater to a wider user base, it is recommended that additional genomes—such as those of *Triticum aestivum*, *Mus musculus*, and Sus scrofa—be included in the benchmarks. This would better validate the tool's robustness across species with varying genome complexities.
Response:
Thanks. We fully agree with this suggestion. Accordingly, we have supplemented the benchmarks with analyses based on another four representative species: common wheat (*Triticum aestivum*, IWGSC CS refseq v2.1), house mouse (*Mus musculus*, GRCm39), wild boar (*Sus scrofa*, Sscrofa11.1), and chicken (*Gallus gallus*, GRCg7b). The results consistently demonstrate the strong performance advantages of *GFFx* across these additional species. The corresponding results have been added to the revision.

2. While the 20-kb interval length used in the region-based retrieval benchmarks is biologically relevant, corresponding to typical gene sizes, it does not fully capture the diversity of genomic query scenarios. To comprehensively assess GFFx's performance across diverse genomic contexts, it is suggested that supplementary benchmarks be conducted using interval lengths of 10 kb and 100 kb. This would help validate the tool's robustness across varying interval scales, which is critical for its practical utility in diverse research workflows.
Response:
We thank the reviewer for this valuable suggestion and fully agree with the point raised. To address it, we supplemented the benchmarks by using intervals ranging from 2.5 kbp to 160 kbp. The results indicate that both runtime and memory usage of

GFFx do increase with larger interval sizes, but the increase is very modest. Specifically, for each doubling of interval size, runtime and memory usage increase by approximately 15-17%. These results have been incorporated into the revised manuscript.

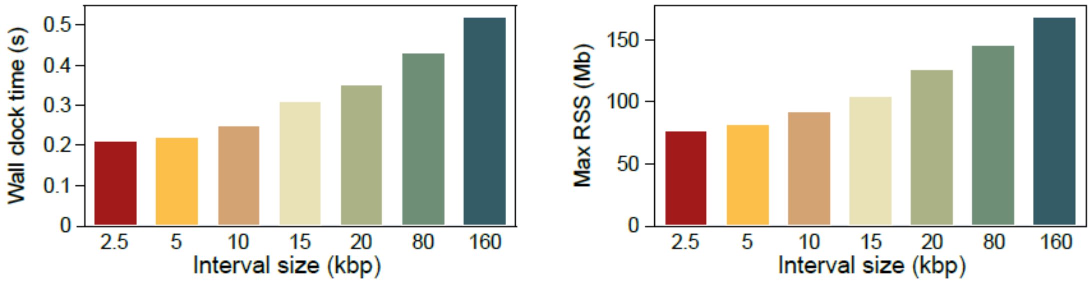

Fig. 3c&3d Median wall-clock time and RSS for extracting 100,000 random intervals with sizes ranging from 2.5 to 160 kbp. Data represent the median of 100 replicate runs. Tools are ordered left-to-right by increasing median wall-clock time on hg38.

3. To further broaden the software's applicability, it is recommended to incorporate an additional functionality that enables the extraction of the number of reads covering specific intervals from BAM files based on positional information derived from GFF3 files, thereby facilitating the calculation of sequencing depth. This feature would be analogous to the functionality provided by `bedtools coverage`, enhancing GFFx's utility in integrating genome annotation data with sequencing read coverage analyses.

Response:

We thank the reviewer for this insightful comment, which is fully aligned with our own development plan. In fact, the implementation of this functionality had already been initiated as part of the ongoing development of the next version, and during the current manuscript revision we incorporated a preliminary coverage profiling module into *GFFx* and added a comparison with the widely used tool *bedtools*. Although the module has not yet undergone extensive optimization, the current implementation already demonstrates clear advantages over *bedtools coverage*. In particular, when using sorted BAM files as input, the new coverage subcommands for breadth and depth calculation are 6.93- to 14.04-fold faster than *bedtools*, while also consuming substantially less memory.

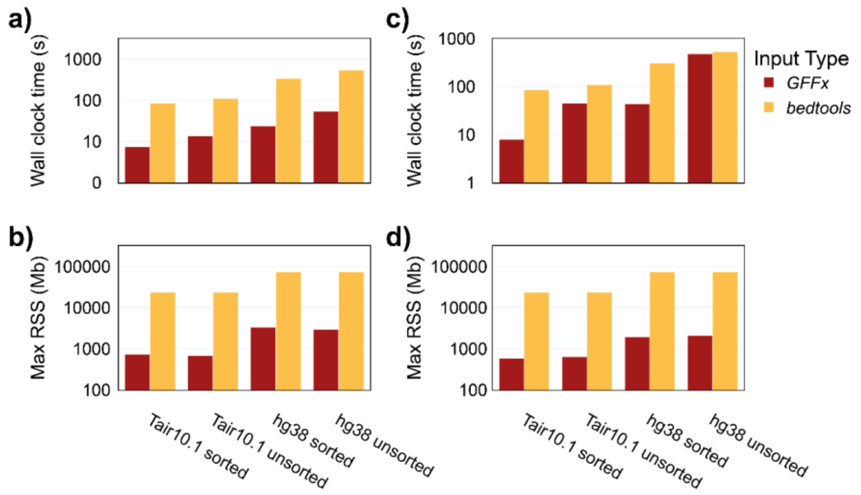

Fig S2. Comparison of coverage profiling performance between GFFx and bedtools. (a) Median wall-clock time (log scale) for quantifying coverage breadth over Tair10.1 (Arabidopsis thaliana) and hg38 (Homo sapiens) genome annotations. (b) Maximum resident set size (RSS, log scale) for quantifying breadth over genome annotations. (c) Median wall-clock time (log scale) for quantifying coverage depth over Tair10.1 and hg38 genome annotations. (d) Maximum resident set size (RSS, log scale) for quantifying depth over genome annotations.

Reviewer #2:

This paper describes GFFx, a new fast and efficient toolkit for working with GFF files. The tool describes a notable advance over current state of the art, and the manuscript overall is well-written. I have only the following minor suggestions for consideration:

Response:

Thanks for the positive comments of our work. We have addressed all suggestions as detailed below.

* In figure S1 and the corresponding discussion, the authors test GFFx on 4 different GFF annotation databases of differing sizes, and differences between the performance is attributed solely to the different dataset sizes. The authors should consider subsetting the largest annotation database (hg38) to more smoothly track how performance and memory use vary with annotation database size, and to confirm there are no organism-specific effects that could underlie the observed differences.

Response:

We thank the reviewer for this constructive suggestion. Following the recommendation, we down-sampled the largest annotation database (hg38) to multiple sizes and repeated the benchmarks. The new results show smooth scaling of runtime and memory usage with dataset size, and within hg38 the relative advantage of *GFFx*

over *gffutils* remained stable across all dataset sizes. By contrast, the variation observed in the cross-organism benchmarks is more likely attributable to differences in annotation complexity rather than dataset size alone. These additional analyses strengthen the conclusion that within a single organism performance is primarily driven by dataset size, while highlighting that the differences across species may reflect more complex biological and annotation-related factors. We note that further studies using a broader range of genomes and annotation styles will be needed to fully disentangle these influences.

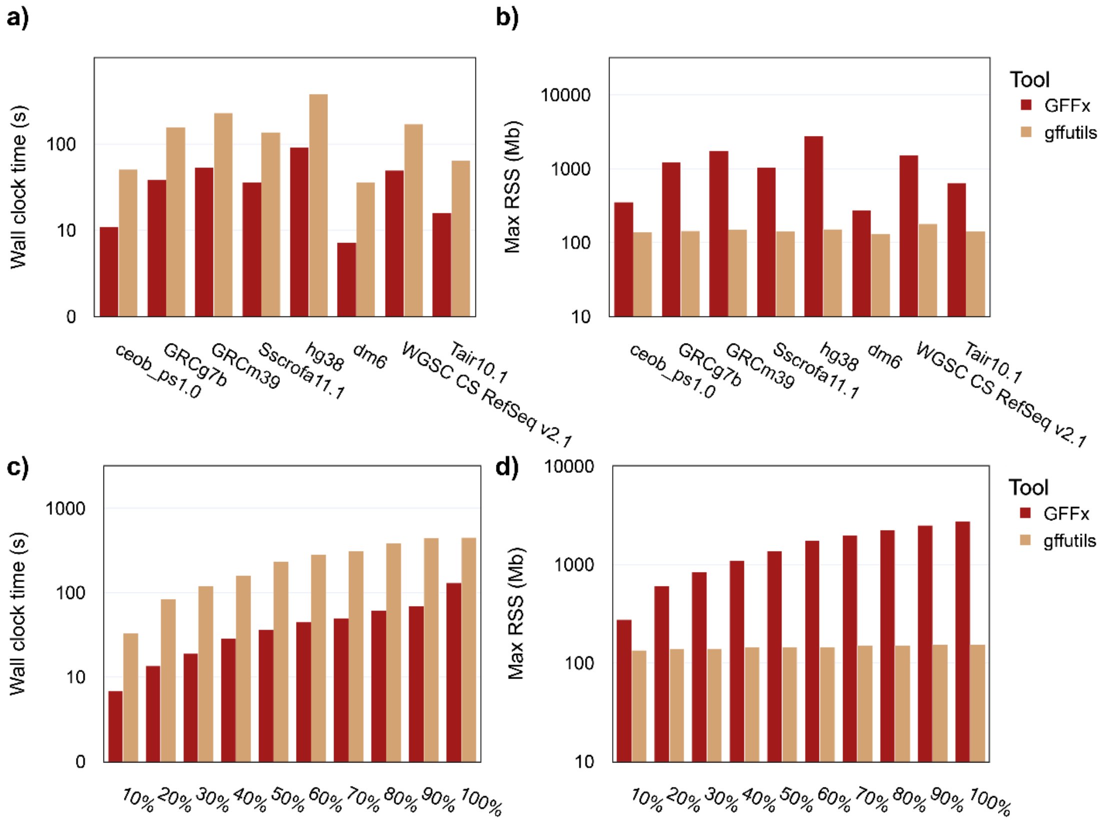

Fig. S1 Comparison of preprocessing performance between *GFFx* and *gfftuils*. (**a**) Median wall-clock time (log scale) on different datasets using GFFx (red) and gffutils (brown). (**b**) Maximum resident set size (RSS, log scale), a measure of peak memory consumption, for each tool and dataset. (**c**) Median wall-clock time on hg38 (Homo sapiens) down-sampled datasets (10%–100%). (**d**) Maximum resident set size (RSS) on hg38 down-sampled datasets (10%–100%).

* The authors should consider changing the line charts in figures 2 and 3 to bar charts — I think the line implies a linear relationship between the tools along the x-axis that is not intended.

Response:

We appreciate this helpful suggestion. We have replaced the line charts in Figures 2 and 3 with bar charts, which more appropriately represent the comparison without implying a linear relationship along the x-axis.

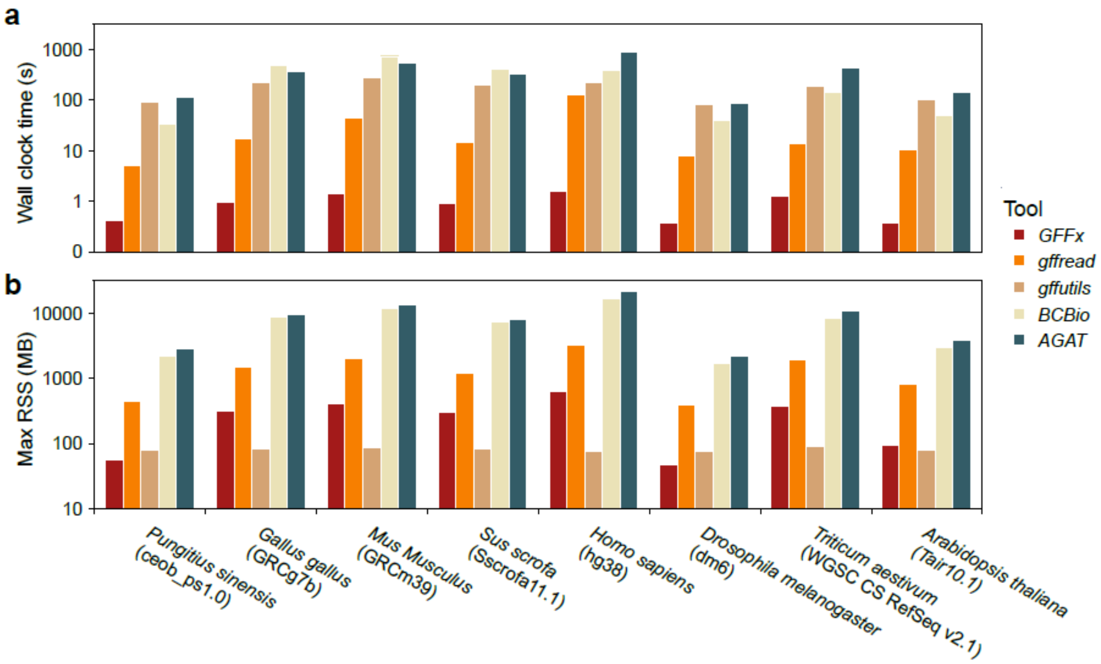

Fig. 2

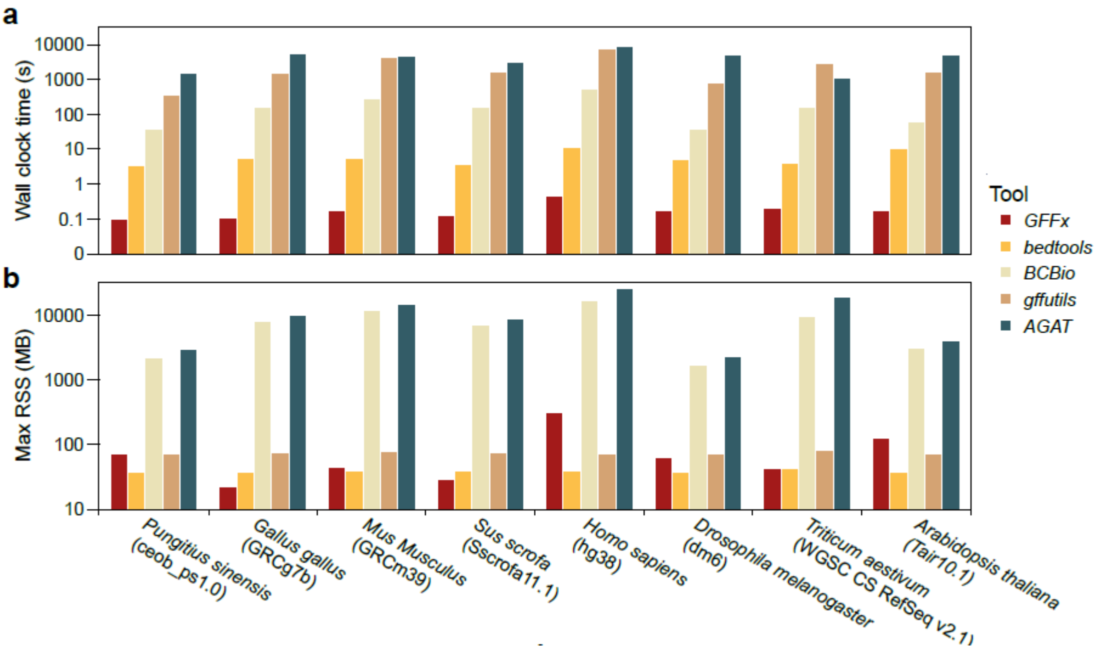

Fig. 3

* For the purposes of benchmarking, the authors used random sampling to extract subsets of the benchmark datasets (e.g., lines 85 and 107). The authors should confirm that the exact same subsets were used when running each tool.

Response:

We confirm that for each replicate, the random subsets of feature identifiers or genomic intervals were generated once and applied consistently across all tools, ensuring that the benchmarking results are directly comparable. We have revised the relevant words to clarify this point.

* In addition to depositing the code and benchmarks on Github, the authors should also deposit snapshots in an archival data repository (like Zenodo).

Response:

We have deposited snapshots of the code and benchmarking data in Zenodo (10.5281/zenodo.17143647) in addition to GitHub, and the corresponding links have been added to the Data Availability section.
