# Supplementary material for: GFFx: A Rust-based suite of utilities for ultra-fast genomic feature extraction
Source: Gigascience. 2025 Oct 23;14:giaf124. doi: 10.1093/gigascience/giaf124 (PMC12548526; doi:10.1093/gigascience/giaf124)
Supplement: giaf124_Authors_Response_To_Reviewer_Comments_Orginal_Submission [file giaf124_authors_response_to_reviewer_comments_orginal_submission.pdf]

Author's Response To Reviewer Comments

Dear Editors and Reviewers,
On behalf of our co-authors, we are pleased to submit a revised version of our manuscript entitled “GFFx: A Rust-Based suite of utilities for ultra-fast genomic feature extraction” for consideration for publication in GigaScience.
We appreciate the time and effort that the editors and the reviewers dedicated to providing feedback on our manuscript and are grateful for the insightful comments on and valuable improvements to our paper. We have incorporated all the suggestions made by the reviewers. Those changes are highlighted within the manuscript. Please see attached point-by-point responses to the reviewers’ comments and concerns.Additionally, we have registered the new software GFFx in both bio.tools and SciCrunch.org and have got an RRID of SCR_027445 and a biotoolsID of “biotools:gffx”.
We believe the revisions have substantially improved the manuscript, and we hope it will now be suitable for publication in GigaScience. We sincerely thank you again for your consideration.
Sincerely,
DongyaWu & Guojie Zhang
Center of Evolutionary & Organismal Biology, Zhejiang University School of Medicine, Hangzhou, 310058, China

Point-by-point response (Manuscript GIGA-D-25-00320)

Reviewer #1:
The overall research appears comprehensive; however, further attention to the tool's capabilities and methodological rigor would strengthen its validity and broader applicability.
Response:
We thank the reviewer for the constructive feedback. We have carefully considered the comments regarding the tool’s capabilities and methodological rigor, and have revised the manuscript accordingly.

1. In the "Performance benchmark in annotation indexing" section, the authors utilized genome annotations from four species (Homo sapiens hg38, Pungitius sinensis ceob_ps_1.0, Drosophila melanogaster dm6, and Arabidopsis thaliana tair10.1) as representatives for benchmarking and subsequent analyses. Nevertheless, a robust GFF processing suite should ideally demonstrate reliability across a broader spectrum of genome types, irrespective of their frequency of use. To enhance the generalizability of GFFx and cater to a wider user base, it is recommended that additional genomes—such as those of Triticum aestivum, Mus musculus, and Sus scrofa—be included in the benchmarks. This would better validate the tool's robustness across species with varying genome complexities.
Response:
Thanks. We fully agree with this suggestion. Accordingly, we have supplemented the benchmarks with analyses based on another four representative species: common wheat (Triticum aestivum, IWGSC CS refseq v2.1), house mouse (Mus musculus, GRCm39), wild boar (Sus scrofa, Sscrofa11.1), and chicken (Gallus gallus, GRCg7b). The results consistently demonstrate the strong performance advantages of GFFx across these additional species. The corresponding results have been added to the revision.

2. While the 20-kb interval length used in the region-based retrieval benchmarks is biologically relevant, corresponding to typical gene sizes, it does not fully capture the diversity of genomic query scenarios. To comprehensively assess GFFx's performance across diverse genomic contexts, it is suggested that supplementary benchmarks be conducted using interval lengths of 10 kb and 100 kb. This would help validate the tool's robustness across varying interval scales, which is critical for its practical utility in diverse research workflows.
Response:
We thank the reviewer for this valuable suggestion and fully agree with the point raised. To address it, we supplemented the benchmarks by using intervals ranging from 2.5 kbp to 160 kbp. The results indicate that both runtime and memory usage of GFFx do increase with larger interval sizes, but the increase is very modest. Specifically, for each doubling of interval size, runtime and memory usage increase by

approximately 15-17%. These results have been incorporated into the revised manuscript.

Fig. 3c&3d Median wall-clock time and RSS for extracting 100,000 random intervals with sizes ranging from 2.5 to 160 kbp. Data represent the median of 100 replicate runs. Tools are ordered left-to-right by increasing median wall-clock time on hg38.

3. To further broaden the software's applicability, it is recommended to incorporate an additional functionality that enables the extraction of the number of reads covering specific intervals from BAM files based on positional information derived from GFF3 files, thereby facilitating the calculation of sequencing depth. This feature would be analogous to the functionality provided by `bedtools coverage`, enhancing GFFx's utility in integrating genome annotation data with sequencing read coverage analyses.
Response:
We thank the reviewer for this insightful comment, which is fully aligned with our own development plan. In fact, the implementation of this functionality had already been initiated as part of the ongoing development of the next version, and during the current manuscript revision we incorporated a preliminary coverage profiling module into GFFx and added a comparison with the widely used tool bedtools. Although the module has not yet undergone extensive optimization, the current implementation already demonstrates clear advantages over bedtools coverage. In particular, when using sorted BAM files as input, the new coverage subcommands for breadth and depth calculation are 6.93- to 14.04-fold faster than bedtools, while also consuming substantially less memory.

Fig S2. Comparison of coverage profiling performance between GFFx and bedtools. (a) Median wall-clock time (log scale) for quantifying coverage breadth over Tair10.1 (Arabidopsis thaliana) and hg38 (Homo sapiens) genome annotations. (b) Maximum resident set size (RSS, log scale) for quantifying breadth over genome annotations. (c) Median wall-clock time (log scale) for quantifying coverage depth over Tair10.1 and hg38 genome annotations. (d) Maximum resident set size (RSS, log scale) for quantifying depth over genome annotations.

Reviewer #2:
This paper describes GFFx, a new fast and efficient toolkit for working with GFF files. The tool describes a notable advance over current state of the art, and the manuscript overall is well-written. I have only the following minor suggestions for consideration:
Response:
Thanks for the positive comments of our work. We have addressed all suggestions as detailed below.

* In figure S1 and the corresponding discussion, the authors test GFFx on 4 different GFF annotation databases of differing sizes, and differences between the performance is attributed solely to the different dataset sizes. The authors should consider subsetting the largest annotation database (hg38) to more smoothly track how performance and memory use vary with annotation database size, and to confirm there are no organism-specific effects that could underlie the observed differences.
Response:
We thank the reviewer for this constructive suggestion. Following the recommendation, we down-sampled the largest annotation database (hg38) to multiple sizes and repeated the benchmarks. The new results show smooth scaling of runtime and memory usage with dataset size, and within hg38 the relative advantage of GFFx over gffutils remained stable across all dataset sizes. By contrast, the variation observed in the cross-organism benchmarks is more likely attributable to differences in annotation complexity rather than dataset size alone. These additional analyses strengthen the conclusion that within a single organism performance is primarily driven by dataset size, while highlighting that the differences across species may reflect more complex biological and annotation-related factors. We note that further studies using a broader range of genomes and annotation styles will be needed to fully disentangle these influences.

Fig. S1 Comparison of preprocessing performance between GFFx and gfftuils. (a) Median wall-clock time (log scale) on different datasets using GFFx (red) and gffutils (brown). (b) Maximum resident set size (RSS, log scale), a measure of peak memory consumption, for each tool and dataset. (c) Median wall-clock

time on hg38 (Homo sapiens) down-sampled datasets (10%–100%). (d) Maximum resident set size (RSS) on hg38 down-sampled datasets (10%–100%).

* The authors should consider changing the line charts in figures 2 and 3 to bar charts — I think the line implies a linear relationship between the tools along the x-axis that is not intended.
Response:
We appreciate this helpful suggestion. We have replaced the line charts in Figures 2 and 3 with bar charts, which more appropriately represent the comparison without implying a linear relationship along the x-axis.

Fig. 2

Fig. 3

* For the purposes of benchmarking, the authors used random sampling to extract subsets of the benchmark datasets (e.g., lines 85 and 107). The authors should confirm that the exact same subsets were used when running each tool.
Response:
We confirm that for each replicate, the random subsets of feature identifiers or genomic intervals were generated once and applied consistently across all tools, ensuring that the benchmarking results are directly comparable. We have revised the relevant words to clarify this point.

* In addition to depositing the code and benchmarks on Github, the authors should also deposit snapshots in an archival data repository (like Zenodo).
Response:
We have deposited snapshots of the code and benchmarking data in Zenodo (10.5281/zenodo.17143647) in addition to GitHub, and the corresponding links have been added to the Data Availability section.

Editorial Manager®

Cookie Preference Center

We use cookies which are necessary to make our site work. We may also use additional cookies to analyze, improve and personalize our content and your digital experience. For more information, see our Cookie Policy.

You may choose not to allow some types of cookies. However, blocking some types may impact your experience of our site and the services we are able to offer. See the different category headings below to find out more or change your settings.

Allow all

Manage Consent Preferences

Strictly Necessary Cookies

Always active

These cookies are necessary for the website to function and cannot be switched off in our systems. They are usually only set in response to actions made by you which amount to a request for services, such as setting your privacy preferences, logging in or filling in forms. You can set your browser to block or alert you about these cookies, but some parts of the site will not then work.

Functional Cookies

These cookies enable the website to provide enhanced functionality and personalisation. They may be set by us or by third party providers whose services we have added to our pages. If you do not allow these cookies then some or all of these services may not function properly.

Performance Cookies

These cookies allow us to count visits and traffic sources so we can measure and improve the performance of our site. They help us to know which pages are the most and least popular and see how visitors move around the site.

Targeting Cookies

These cookies may be set through our site by our advertising partners. They may be used by those companies to build a profile of your interests and show you relevant adverts on other sites. If you do not allow these cookies, you will experience less targeted advertising.

Cookie List

Clear

Apply Cancel

Consent Leg.Interest

Confirm my choices

Powered by onetrust
