# Supplementary material for: GFFx: A Rust-based suite of utilities for ultra-fast genomic feature extraction
Source: Gigascience. 2025 Oct 23;14:giaf124. doi: 10.1093/gigascience/giaf124 (PMC12548526; doi:10.1093/gigascience/giaf124)
Supplement: giaf124_Reviewer_1_Report_Original_Submission — Xingtan Zhang -- 8/24/2025 [file giaf124_reviewer_1_report_original_submission.pdf]

Reviewer Report

Title: GFFx: A Rust-based suite of utilities for ultra-fast genomic feature extraction

Version: Original Submission **Date:** 8/24/2025

Reviewer name: Xingtan Zhang

Reviewer Comments to Author:

The overall research appears comprehensive; however, further attention to the tool's capabilities and methodological rigor would strengthen its validity and broader applicability.1. In the "Performance benchmark in annotation indexing" section, the authors utilized genome annotations from four species (Homo sapiens hg38, Pungitius sinensis ceob_ps_1.0, Drosophila melanogaster dm6, and Arabidopsis thaliana tair10.1) as representatives for benchmarking and subsequent analyses. Nevertheless, a robust GFF processing suite should ideally demonstrate reliability across a broader spectrum of genome types, irrespective of their frequency of use. To enhance the generalizability of GFFx and cater to a wider user base, it is recommended that additional genomesâ€"such as those of Triticum aestivum, Mus musculus, and Sus scrofaâ€"be included in the benchmarks. This would better validate the tool's robustness across species with varying genome complexities.2. While the 20-kb interval length used in the region-based retrieval benchmarks is biologically relevant, corresponding to typical gene sizes, it does not fully capture the diversity of genomic query scenarios. To comprehensively assess GFFx's performance across diverse genomic contexts, it is suggested that supplementary benchmarks be conducted using interval lengths of 10 kb and 100 kb. This would help validate the tool's robustness across varying interval scales, which is critical for its practical utility in diverse research workflows.3. To further broaden the software's applicability, it is recommended to incorporate an additional functionality that enables the extraction of the number of reads covering specific intervals from BAM files based on positional information derived from GFF3 files, thereby facilitating the calculation of sequencing depth. This feature would be analogous to the functionality provided by `bedtools coverage`, enhancing GFFx's utility in integrating genome annotation data with sequencing read coverage analyses.

Level of Interest

Please indicate how interesting you found the manuscript: Choose an item.

Quality of Written English

Please indicate the quality of language in the manuscript: Choose an item.

Declaration of Competing Interests

Please complete a declaration of competing interests, considering the following questions:

- Have you in the past five years received reimbursements, fees, funding, or salary from an organisation that may in any way gain or lose financially from the publication of this manuscript, either now or in the future?
- Do you hold any stocks or shares in an organisation that may in any way gain or lose financially from the publication of this manuscript, either now or in the future?
- Do you hold or are you currently applying for any patents relating to the content of the manuscript?
- Have you received reimbursements, fees, funding, or salary from an organization that holds or has applied for patents relating to the content of the manuscript?
- Do you have any other financial competing interests?
- Do you have any non-financial competing interests in relation to this paper?

If you can answer no to all of the above, write 'I declare that I have no competing interests' below. If your reply is yes to any, please give details below.

I declare that I have no competing interests

I agree to the open peer review policy of the journal. I understand that my name will be included on my report to the authors and, if the manuscript is accepted for publication, my named report including any attachments I upload will be posted on the website along with the authors' responses. I agree for my report to be made available under an Open Access Creative Commons CC-BY license (http://creativecommons.org/licenses/by/4.0/). I understand that any comments which I do not wish to be included in my named report can be included as confidential comments to the editors, which will not be published.

Choose an item.

To further support our reviewers, we have joined with Publons, where you can gain additional credit to further highlight your hard work (see: https://publons.com/journal/530/gigascience). On publication of this paper, your review will be automatically added to Publons, you can then choose whether or not to claim your Publons credit. I understand this statement.

Yes Choose an item.
