# Supplementary material for: GFFx: A Rust-based suite of utilities for ultra-fast genomic feature extraction
Source: Gigascience. 2025 Oct 23;14:giaf124. doi: 10.1093/gigascience/giaf124 (PMC12548526; doi:10.1093/gigascience/giaf124)
Supplement: giaf124_Reviewer_2_Report_Original_Submission — Andrew Su -- 9/8/2025 [file giaf124_reviewer_2_report_original_submission.pdf]

Reviewer Report

Title: GFFx: A Rust-based suite of utilities for ultra-fast genomic feature extraction

Version: Original Submission **Date:** 9/8/2025

Reviewer name: Andrew Su

Reviewer Comments to Author:

This paper describes GFFx, a new fast and efficient toolkit for working with GFF files. The tool describes a notable advance over curent state of the art, and the manuscript overall is well-written. I have only the following minor suggestions for consideration:* In figure S1 and the corresponding discussion, the authors test GFFx on 4 different GFF annotation databases of differing sizes, and differences between the performance is attributed solely to the different dataset sizes. The authors should consider subsetting the largest annotation database (hg38) to more smoothly track how performance and memory use vary with annotation database size, and to confirm there are no organism-specific effects that could underlie the observed differences.* The authors should consider changing the line charts in figures 2 and 3 to bar charts â€” I think the line implies a linear relationship between the tools along the x-axis that is not intended. * For the purposes of benchmarking, the authors used random sampling to extract subsets of the benchmark datasets (e.g., lines 85 and 107). The authors should confirm that the exact same subsets were used when running each tool.* In addition to depositing the code and benchmarks on Github, the authors should also deposit snapshots in an archival data repository (like Zenodo).

Level of Interest

Please indicate how interesting you found the manuscript: Choose an item.

Quality of Written English

Please indicate the quality of language in the manuscript: Choose an item.

Declaration of Competing Interests

Please complete a declaration of competing interests, considering the following questions:

- Have you in the past five years received reimbursements, fees, funding, or salary from an organisation that may in any way gain or lose financially from the publication of this manuscript, either now or in the future?
- Do you hold any stocks or shares in an organisation that may in any way gain or lose financially from the publication of this manuscript, either now or in the future?
- Do you hold or are you currently applying for any patents relating to the content of the manuscript?

- Have you received reimbursements, fees, funding, or salary from an organization that holds or has applied for patents relating to the content of the manuscript?
- Do you have any other financial competing interests?
- Do you have any non-financial competing interests in relation to this paper?

If you can answer no to all of the above, write 'I declare that I have no competing interests' below. If your reply is yes to any, please give details below.

I declare that I have no competing interests

I agree to the open peer review policy of the journal. I understand that my name will be included on my report to the authors and, if the manuscript is accepted for publication, my named report including any attachments I upload will be posted on the website along with the authors' responses. I agree for my report to be made available under an Open Access Creative Commons CC-BY license (http://creativecommons.org/licenses/by/4.0/). I understand that any comments which I do not wish to be included in my named report can be included as confidential comments to the editors, which will not be published.

Choose an item.

To further support our reviewers, we have joined with Publons, where you can gain additional credit to further highlight your hard work (see: https://publons.com/journal/530/gigascience). On publication of this paper, your review will be automatically added to Publons, you can then choose whether or not to claim your Publons credit. I understand this statement.

Yes Choose an item.
